# Natural Products as Promising Pharmacological Agents Against Cancer: A Holistic Overview of Their Anti-Cancer Mechanisms of Action of the Last Five Years

**DOI:** 10.3390/ph19060910

**Published:** 2026-06-09

**Authors:** Sousana K. Papadopoulou, Efthymios Poulios, Agathi Pritsa, Evmorfia Psara, Athanasios Migdanis, Constantinos Giaginis

**Affiliations:** 1 Department of Nutritional Sciences and Dietetics, International Hellenic University, Nea Moudania, 57001 Thessaloniki, Greece; souzpap@gmail.com; 2 Department of Food Science and Nutrition, University of the Aegean, 81400 Myrina, Greece; epoulios@aegean.gr (E.P.); fnsd21013@fns.aegean.gr (E.P.); 3 Nutrition and Dietetics Department, University of Thessaly, Argonafton 1C, 42132 Trikala, Greece; amigdanis@gmail.com; 4 Nutrition and Dietetics Department, University General Hospital of Larissa, Viopolis Mezourlo, 41110 Larissa, Greece

**Keywords:** natural products, cancer, drug design and discovery, pharmacological agents, anticancer activity

## Abstract

**Background/Objectives**: Natural products have long been regarded as a cornerstone in the discovery and development of novel therapeutic agents. Accumulating evidence indicates that natural products represent promising pharmacological candidates for cancer treatment. This review provides a holistic overview of novel identified natural products as a continuing source of bioactive compounds, with particular emphasis on recent advances and their applications in anticancer therapy over the past five years. **Methods**: A literature search was conducted using PubMed, Scopus, and Web of Science to identify relevant studies published within the past five years. Predefined keywords and Boolean operators (e.g., “natural products”, “anticancer”, “drug discovery”, “secondary metabolites”, “signaling pathways”, “epigenetics”) were applied, with search strategies adapted to each database. Eligible studies included original research articles and reviews reporting on newly identified natural products with anticancer activity, with emphasis on chemical diversity, biological effects, and molecular mechanisms of action. Additional references were identified through manual screening of bibliographies. The selected literature was evaluated using a qualitative, interpretative approach consistent with narrative review methodology, and findings were critically synthesized and thematically organized. **Results**: Growing evidence indicates that multiple newly identified natural products target mitochondrial metabolism and interact with alternative tubulin binding sites, thereby highlighting their potential as anticancer agents. In addition, emerging compounds have been shown to affect DNA integrity and transcriptional regulation, while also acting as systems-level modulators of key oncogenic signaling pathways, including PI3K/Akt, NF-κB, and MAPK. Recent studies further demonstrate that natural products can modulate multiple layers of epigenetic regulation, including DNA methylation, histone acetylation, histone methylation, and non-coding RNA networks. **Conclusions**: Current evidence supports the concept that natural products primarily function as multi-target biological modulators rather than classical single-target inhibitors in cancer biology. A persistent challenge remains the translational gap between preclinical efficacy and clinical application, as the majority of naturally derived candidate compounds remain confined to in vitro or early in vivo validation. Future progress will therefore depend on systematically aligning the multi-target pharmacology of natural products with defined cancer vulnerabilities and clinically actionable therapeutic strategies.

## 1. Introduction

Natural products have historically constituted a cornerstone of drug discovery, providing an exceptional diversity of structurally complex and biologically active molecules. These compounds, derived from plants, microorganisms, marine organisms, and fungi, are shaped by evolutionary pressures that optimize their ecological functions, including defense, interspecies communication, and competitive interactions [1,2]. This evolutionary refinement has conferred upon natural products a high degree of affinity for biological macromolecules, rendering them particularly valuable as pharmacological agents. It is estimated that a substantial proportion of approved drugs are either natural products, natural product derivatives, or compounds inspired by natural scaffolds, underscoring their sustained relevance in medicinal chemistry [3,4].

The contribution of natural products is especially prominent in oncology, where they have yielded some of the most effective chemotherapeutic agents [5,6]. Compounds such as paclitaxel, doxorubicin, and camptothecin derivatives exemplify the diverse mechanisms through which natural products exert anticancer activity, including microtubule stabilization, DNA intercalation, and topoisomerase inhibition [6,7]. Beyond these canonical mechanisms, recent evidence indicates that naturally derived compounds modulate a broad spectrum of cellular processes, including apoptosis, autophagy, angiogenesis, immune evasion, and epigenetic regulation [4,5,6,7]. Their capacity to simultaneously engage multiple molecular targets is particularly advantageous in oncology, where signaling redundancy and adaptive resistance frequently compromise the efficacy of single-target therapeutics [4,6,7].

From a structural perspective, naturally occurring compounds occupy a unique and highly complex chemical space that is difficult to replicate using conventional synthetic libraries. Features such as high stereochemical density, rigid three-dimensional architectures, macrocyclic frameworks, and diverse functional group distributions contribute to their enhanced binding specificity and biological activity [1,4]. Major classes, including alkaloids, polyketides, terpenoids, and non-ribosomal peptides, exhibit remarkable biosynthetic diversity, often arising from hybrid enzymatic pathways that integrate multiple biosynthetic systems [1,4,5]. This structural richness not only underpins their pharmacological relevance but also provides valuable scaffolds for the development of novel therapeutic agents through semi-synthetic modification and structure-activity relationship (SAR) optimization [8,9].

Recent technological advances have considerably revitalized natural product research and expanded the scope of compound discovery. High-resolution analytical platforms, including nuclear magnetic resonance (NMR) spectroscopy, high-resolution mass spectrometry (HRMS), and cryo-electron microscopy (cryo-EM), have enabled rapid and precise structural elucidation of complex molecules, even from minute quantities [10,11]. In parallel, genome mining and metagenomic sequencing have transformed the identification of biosynthetic gene clusters (BGCs), enabling the prediction and discovery of novel natural products from previously uncultivable microorganisms [12,13]. Computational platforms such as antiSMASH and PRISM have further facilitated the annotation and prioritization of these gene clusters, significantly accelerating the natural product discovery pipeline [13,14].

Concurrently, synthetic biology and metabolic engineering have emerged as powerful strategies to overcome longstanding limitations in natural product research, particularly those related to supply and scalability. Heterologous expression of biosynthetic pathways in engineered microbial hosts, such as Escherichia coli and Streptomyces species, has enabled the sustainable production of complex natural products and their analogues [3,4]. Advances in total synthesis and semi-synthesis have further expanded access to scarce natural compounds while enabling systematic optimization of their pharmacological properties [15,16,17]. Moreover, the integration of artificial intelligence (AI) and machine learning (ML) is increasingly transforming the field by enabling the prediction of molecular structure, bioactivity, and synthetic feasibility, thereby streamlining the identification of promising drug candidates [15,16,17].

Importantly, marine ecosystems and extremophilic environments represent particularly rich and underexplored sources of novel natural products [18,19]. Marine organisms such as sponges, tunicates, and cyanobacteria produce chemically unique metabolites, often characterized by halogenation patterns, uncommon heterocyclic motifs, and complex macrocyclic architectures [18,19]. Similarly, extremophiles inhabiting deep-sea hydrothermal vents, polar regions, and hypersaline environments have evolved distinctive metabolic pathways that generate unprecedented chemical scaffolds [20,21]. These environments have contributed significantly to the discovery of novel anticancer agents and remain a major frontier in natural product research [20,21].

Research Gap and Need for the Study

Despite substantial advances in analytical technologies, biosynthetic pathway elucidation, and computational drug discovery, the field of natural product-based anticancer research remains fragmented across chemical, biological, and translational domains. Existing literature is often compartmentalized, focusing either on isolated compound classes, specific biological mechanisms, or individual technological platforms, rather than providing an integrated synthesis of these interconnected dimensions. Furthermore, recent rapid expansions in genome mining, AI-driven discovery tools, and marine-derived compound research have not yet been comprehensively consolidated in a manner that links structural diversity, mechanistic anticancer activity, and translational potential.

In addition, while numerous reviews address specific subsets of natural products or individual mechanisms of action, there is a lack of updated, integrative narrative synthesis that simultaneously captures emerging natural compounds, evolving biosynthetic insights, and modern discovery technologies within a unified oncological framework. This fragmentation limits the ability to identify overarching trends, shared mechanistic signatures, and translational opportunities across diverse natural product classes.

Accordingly, there is a clear need for a comprehensive and conceptually integrative narrative review that consolidates recent advances in natural product discovery, mechanistic anticancer activity, and enabling technologies. Such synthesis is essential to bridge disciplinary silos, highlight emerging research trajectories, and guide future drug development efforts in oncology.

Aim of the Review

This narrative review aims to provide a comprehensive and up-to-date holistic overview of natural products as a continuing source of novel bioactive compounds, with a particular focus on recent discoveries and their applications in anticancer therapy over the last five years. Emphasis is placed on newly identified compounds, emerging biological sources, advances in biosynthetic understanding, and innovative technologies shaping the future of natural product-based drug discovery. By highlighting current trends, methodological advances, and persistent challenges, this review seeks to underscore the enduring and evolving relevance of natural products in the development of next-generation anticancer therapeutics.

## 2. Results

### 2.1. Recent Natural Products with Anticancer Properties over the Last Five Years

#### 2.1.1. Basic Principles

**Figure 1:**
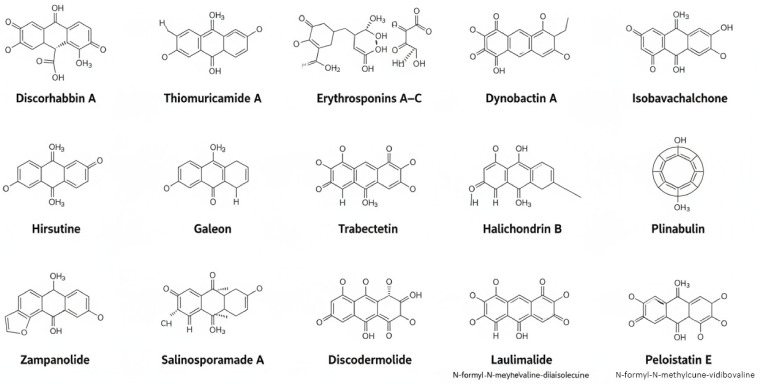
The most recently identified natural products studied over the last five years.

Natural products have long constituted a fundamental source of anticancer agents, with a substantial proportion of approved chemotherapeutic drugs derived directly or indirectly from natural scaffolds [6,7]. Despite significant developments in synthetic chemistry, natural products continue to be uniquely valuable due to their structural complexity, stereochemical diversity, and evolutionary optimization for interactions with biological macromolecules, particularly proteins [8,9,10]. In oncology, these compounds have consistently contributed both clinically approved drugs and novel lead structures targeting key hallmarks of cancer, including sustained proliferative signaling, angiogenesis, resistance to apoptosis, and metabolic reprogramming [6,7,8]. The most recently identified natural products studied over the past five years are presented in Figure 1.

In the last few years, the discovery of anticancer natural products has increasingly shifted toward marine biodiversity, microbial genome mining, and extremophile-derived metabolites, supported by advances in high-throughput omics technologies and AI-assisted drug discovery approaches [13,15,16,17]. This transition has resulted in a substantial expansion of accessible chemical space and facilitated the identification of previously uncharacterized biosynthetic pathways [12,18,19].

#### 2.1.2. Marine-Derived Anticancer Natural Products

Marine ecosystems continue to dominate recent efforts in anticancer natural product discovery due to their exceptional chemical diversity and the strong ecological selection pressures that drive unique biosynthetic adaptations [21,22,23]. Between 2020 and 2024, marine-derived compounds have been extensively highlighted as a major and productive pipeline for anticancer drug development [24,25,26].

Currently, the clinically relevant marine-derived anticancer agents display marked mechanistic and structural diversity. More to the point, trabectedin binds the DNA minor groove at GC-rich sequences, inducing DNA bending and disrupting transcription and DNA repair, thereby primarily targeting genomic stability and transcriptional regulation. In contrast, eribulin, a synthetic analogue of halichondrin B, inhibits microtubule polymer growth at the plus ends of tubulin, leading to irreversible mitotic arrest and vascular remodeling, representing a distinct cytoskeletal-targeting strategy [21,22,23]. Expanding this mechanistic spectrum, diketopiperazines such as plinabulin exhibit both microtubule-destabilizing and immunomodulatory activity, including dendritic cell activation, thereby integrating direct tumor cytotoxicity with tumor microenvironment modulation [27,28,29].

More structurally complex sponge- and tunicate-derived metabolites, including macrolides, polyketides, and peptide–polyketide hybrids, further broaden this landscape by acting through apoptosis induction, mitochondrial dysfunction, ROS generation, and angiogenesis inhibition. These compounds often share features such as extensive stereochemical complexity, frequent halogenation, and hybrid biosynthetic architectures, which enhance target affinity and biological potency while enabling multi-pathway activity [19,25,28,29,30,31]. Collectively, comparative analysis highlights a continuum of marine-derived anticancer mechanisms ranging from highly specific DNA- or microtubule-targeting agents to multifunctional compounds acting on apoptosis, metabolism, and tumor angiogenesis, underscoring the pharmacological versatility of marine natural product scaffolds.

#### 2.1.3. Microbial and Cyanobacterial Anticancer Natural Products

Microorganisms remain among the most prolific sources of bioactive secondary metabolites, particularly in the context of recent advances in genome mining and metagenomic approaches [12,13,14]. Cyanobacterial metabolites, in particular, have attracted increasing attention in oncology due to their ability to modulate cellular metabolism and mitochondrial function.

A notable example is nocuolin A, a cyanobacterial alkaloid that induces apoptosis through inhibition of mitochondrial oxidative phosphorylation, causing energetic collapse and representing a metabolic mode of cytotoxicity distinct from DNA- or microtubule-targeting agents [3,4,19]. In contrast, ribosomally synthesized and post-translationally modified peptides (RiPPs) such as dynobactin A act through disruption of essential protein complexes and protein–protein interactions, representing a structurally constrained class of protein-function-targeting natural products with emerging anticancer relevance [22,24].

Salinosporamide A and related analogues, derived from marine actinomycetes, function as potent irreversible proteasome inhibitors, leading to accumulation of misfolded proteins, disruption of protein homeostasis, and activation of apoptosis through endoplasmic reticulum stress pathways. In contrast, other actinomycete-derived macrolides primarily exert cytotoxicity through DNA intercalation, transcriptional inhibition, or indirect modulation of cell-cycle progression, representing a more classical class of nucleic acid-associated cytotoxins [8,9].

Comparatively, these microbial natural product classes delineate three principal anticancer strategies: (i) metabolic disruption (nocuolin A targeting mitochondrial respiration), (ii) protein interaction and complex disruption (RiPPs such as dynobactin A), and (iii) protein degradation or DNA-targeting cytotoxicity (salinosporamide A and actinomycete macrolides). Conclusively, this diversity highlights the evolutionary breadth of microbial biosynthetic systems and underscores their importance as a source of structurally and mechanistically distinct scaffolds for anticancer drug discovery.

#### 2.1.4. Plant-Derived Anticancer Natural Products

Although plant-derived natural products have historically yielded several major anticancer drugs, recent research has increasingly focused on mechanistic elucidation, structural optimization of derivatives, and pathway-specific modulation rather than the identification of entirely new clinical drug candidates [4,9,24].

Compounds such as isobavachalcone, a prenylated chalcone, primarily exert anticancer effects through inhibition of NF-κB signaling, resulting in reduced inflammatory transcriptional activity and suppression of tumor cell proliferation. In comparative terms, this mechanism positions isobavachalcone within a class of transcription factor-modulating phytochemicals, which act upstream of multiple survival and proliferation pathways [28,29]. In contrast, hirsutine, an indole alkaloid, induces apoptosis predominantly through inhibition of the PI3K/Akt survival signaling cascade, coupled with activation of reactive oxygen species (ROS)-mediated cellular stress responses, thereby integrating kinase pathway suppression with redox imbalance-driven cytotoxicity [10,31].

Recent phytochemical investigations further expand this mechanistic spectrum through flavonoids and xanthone derivatives, which display broader polypharmacological activity across multiple cancer types, including breast, liver, and lung cancers [27,28]. These compounds commonly act through epigenetic modulation (e.g., histone acetylation and DNA methylation regulation), kinase inhibition (targeting MAPK/PI3K-related pathways), and cell-cycle arrest at G0/G1 or G2/M phases, reflecting a more multi-target regulatory mode of action compared with single-pathway inhibitors [27,28]. Comparative analysis of these plant-derived compounds highlights a mechanistic continuum ranging from specific signaling-axis inhibition (NF-κB, PI3K/Akt) to multi-pathway epigenetic and cell-cycle regulation, underscoring the structural diversity and pharmacological versatility of phytochemical scaffolds in anticancer drug discovery.

#### 2.1.5. Fungal and Endophytic Anticancer Metabolites

Fungal secondary metabolites represent a structurally diverse source of anticancer agents spanning both direct cytotoxic compounds and immunomodulatory macromolecules. Endophytic fungal metabolites primarily induce apoptosis through caspase activation, mitochondrial dysfunction, and oxidative stress, reflecting a mechanism centered on mitochondrial-mediated cell death [26,32]. In contrast, marine-derived fungal metabolites, including polyketides and nitrogen-containing compounds, exhibit broader activity profiles, acting through enzyme inhibition, redox imbalance, cell-cycle arrest, and occasional DNA or signaling pathway interactions, indicating greater mechanistic diversity and multi-target effects [27,29,30].

Distinct from these low-molecular-weight cytotoxins, fungal polysaccharides exert anticancer effects indirectly via immunomodulation, enhancing host immune responses such as macrophage and T-cell activation rather than directly killing tumor cells [33,34,35]. Comparative analysis of fungal-derived natural products reveals three principal anticancer strategies: (i) direct mitochondrial and apoptosis induction (endophytic metabolites), (ii) broad-spectrum cytotoxic and signaling disruption (marine-derived fungal polyketides and nitrogenous compounds), and (iii) immune-mediated tumor suppression (fungal polysaccharides). This mechanistic diversity underscores fungi as a uniquely versatile source of anticancer agents spanning both cell-autonomous cytotoxicity and host-directed immunomodulation.

#### 2.1.6. Emerging Mechanistic Trends in Natural Products

Across all major natural product classes, several convergent mechanistic trends have been identified, as summarized in Table 1. Together, these observations indicate a paradigm shift from conventional single-target cytotoxicity toward multi-target, pathway-integrated, and systems-level anticancer strategies.

Collectively, the period from 2021 to 2026 has witnessed a marked resurgence in anticancer natural product discovery, driven by technological innovation and the exploration of previously underexploited ecological niches. Marine organisms, microorganisms, plants, and fungi continue to yield structurally novel compounds with diverse and often multifaceted mechanisms of action. Notably, the field is progressively shifting away from conventional cytotoxic agents toward therapeutics targeting metabolic regulation, immune modulation, and oncogenic signaling networks, reflecting a broader paradigm shift in natural product-based drug discovery. Ongoing integration of synthetic biology, artificial intelligence-driven screening platforms, and multi-omics technologies will be essential for translating these discoveries into clinically viable anticancer therapeutics.

### 2.2. Natural Products Targeting Mitochondrial Metabolism in Cancer

#### 2.2.1. Mitochondrial Metabolism as a Therapeutic Vulnerability in Cancer

Mitochondria have re-emerged as central regulators of cancer cell survival, extending beyond ATP production to include the maintenance of redox homeostasis, biosynthetic flux, and apoptotic signaling. Cancer cells frequently retain or reprogram oxidative phosphorylation (OXPHOS), particularly within metastatic, drug-resistant, and stem-like populations, thereby rendering mitochondrial metabolism a critical therapeutic vulnerability [53,54]. Consequently, natural products that disrupt mitochondrial function—especially those targeting electron transport chain (ETC) complexes—have attracted increasing attention as potential anticancer agents [36,55].

In recent years, research has shifted from broadly defined “mitochondrial toxicants” toward mechanistically characterized mitochondrial modulators, particularly compounds targeting Complex I, mitochondrial membrane potential, and the metabolic coupling between glycolysis and OXPHOS [12,37]. This transition has been facilitated by advances in high-resolution respirometry, metabolomics, and cryo-electron microscopy-based structural validation [12,37].

#### 2.2.2. Complex I Inhibitors and Disruption of Electron Transport Chain Flux

One of the most prominent emerging classes of mitochondrial-targeting natural products comprises inhibitors of mitochondrial Complex I (NADH:ubiquinone oxidoreductase). As the primary entry point for electrons into the ETC, Complex I plays a central role in maintaining NAD^+^/NADH balance and regulating reactive oxygen species (ROS) homeostasis.

Substantial studies highlight that structurally diverse natural product classes—including alkaloids, acetogenins, and polyketides—converge functionally on mitochondrial Complex I inhibition, leading to metabolic collapse in tumor cells [26,56]. Despite this shared target, these scaffolds exhibit notable mechanistic and pharmacological differences. Acetogenins are the most potent and well-characterized inhibitors, binding directly to the ubiquinone-binding site of Complex I, thereby blocking electron transfer, proton translocation, and ATP synthesis with nanomolar potency. This high affinity, however, is associated with a narrow therapeutic index, limiting their clinical applicability [22,38].

In contrast, alkaloids often display more moderate and pleiotropic effects, combining partial Complex I inhibition with additional activities such as ROS induction and signaling pathway modulation, which may enhance anticancer efficacy while potentially improving tolerability [26,56]. Polyketides, on the other hand, tend to exhibit broader multi-target profiles, influencing not only mitochondrial respiration but also redox balance and cell-cycle regulation, reflecting their structural flexibility and biosynthetic diversity [57,58].

Functionally, all three classes induce bioenergetic failure, elevated ROS production, and activation of intrinsic apoptotic pathways, particularly in cancer cells reliant on oxidative phosphorylation under hypoxic or metastatic conditions [57,58]. However, comparative analysis indicates a continuum ranging from highly specific, high-potency mitochondrial blockade (acetogenins) to multi-target metabolic and signaling disruption (alkaloids and polyketides) [26,38,56]. This diversity underscores both the therapeutic promise and the translational challenges of targeting mitochondrial metabolism using natural-product scaffolds.

#### 2.2.3. Cyanobacterial and Microbial Metabolites Targeting Mitochondrial Respiration

Cyanobacteria- and microbe-derived natural products that target mitochondrial function exhibit convergent bioenergetic effects but distinct primary mechanisms and structural features. nocuolin A, an oxadiazine-containing alkaloid, acts as a direct inhibitor of mitochondrial oxidative phosphorylation, reducing respiratory capacity and ATP production, thereby triggering apoptosis through energy depletion-driven stress signaling [59,60,61]. This places nocuolin A within a class of metabolism-focused mitochondrial inhibitors that primarily disrupt cellular bioenergetics.

In contrast, ribosomally synthesized macrocyclic peptides such as dynobactin A operate through a fundamentally different mechanism. Rather than directly inhibiting respiration, these peptides are thought to interfere with protein–membrane or protein–organelle interactions, potentially altering mitochondrial integrity or function indirectly. This represents a protein-interaction-targeting strategy, distinct from the metabolic inhibition seen with nocuolin A, and suggests opportunities for engineered selectivity toward mitochondrial-associated protein complexes [59,60,61].

Meanwhile, microbial polyketides and macrolides derived from Streptomyces and related genera exhibit a broader, downstream mode of action, inducing apoptosis through mitochondrial membrane depolarization, cytochrome c release, and ROS amplification [4,5]. Unlike nocuolin A, which directly impairs respiratory function, or peptides that target protein interactions, these compounds primarily act by destabilizing mitochondrial integrity and enhancing oxidative stress, often as part of a multi-target cytotoxic profile.

#### 2.2.4. Plant-Derived Modulators of Mitochondrial Bioenergetics

Plant-derived natural products continue to act as indirect modulators of mitochondrial function, primarily through upstream signaling pathways rather than direct mitochondrial targeting. Isobavachalcone, a prenylated chalcone, exerts its effects mainly via NF-κB inhibition, leading to increased ROS levels and subsequent mitochondria-dependent apoptosis [62,63]. In contrast, hirsutine, an indole alkaloid, targets the PI3K/Akt survival pathway, resulting in mitochondrial membrane depolarization and caspase activation [64].

Practically, both compounds converge on mitochondrial dysfunction and apoptotic signaling, but differ in their upstream triggers: isobavachalcone primarily modulates inflammatory/transcriptional signaling, whereas hirsutine interferes with cell survival kinase pathways. This highlights a broader trend among plant-derived anticancer agents, where mitochondrial impairment arises as a downstream consequence of signaling pathway disruption, rather than direct inhibition of mitochondrial machinery.

#### 2.2.5. Functional Consequences of Mitochondrial Targeting in Cancer Cells

Across all the natural products, mitochondrial targeting consistently results in a series of convergent anticancer outcomes, as summarized in Table 2. Importantly, mitochondrial targeting appears to be particularly effective in tumor subpopulations that preferentially rely on oxidative metabolism rather than glycolysis, including cancer stem cells and metastatic cells, thereby suggesting a selective therapeutic window [65].

#### 2.2.6. Conclusion, Remarks and Future Perspectives

Current substantial evidence indicates that natural products targeting mitochondrial metabolism constitute a rapidly expanding and mechanistically diverse class of anticancer agents. The most significant developments include Complex I inhibitors, cyanobacterial OXPHOS disruptors, and plant-derived signaling modulators that indirectly compromise mitochondrial integrity [65,66]. Nevertheless, important challenges remain, particularly with respect to target selectivity, systemic toxicity, and pharmacokinetic optimization.

Future progress will likely depend on the integration of structural biology, metabolomics, and artificial intelligence-driven compound prioritization, alongside synthetic biology approaches for scalable and sustainable production. Overall, mitochondrial metabolism represents one of the most promising yet still underexploited therapeutic axes for next-generation natural product-based anticancer drug discovery.

### 2.3. Marine Natural Products Targeting Microtubule Dynamics in Cancer: Emerging Roles of Peptides and Macrolides

#### 2.3.1. Microtubule Dynamics as a Validated Anticancer Vulnerability

Microtubules are dynamic cytoskeletal polymers composed of α/β-tubulin heterodimers that undergo continuous cycles of polymerization and depolymerization. This dynamic instability is essential for mitotic spindle assembly and successful cell division [67,68]. Pharmacological disruption of microtubule dynamics results in mitotic arrest, chromosomal missegregation, and apoptosis, establishing microtubules as one of the most successful and clinically validated targets in anticancer chemotherapy [67,68].

Clinically established agents such as taxanes and vinca alkaloids validate this therapeutic strategy; however, their long-term efficacy is limited by resistance mechanisms, including P-glycoprotein-mediated efflux and β-tubulin mutations [69,70]. These limitations have intensified interest in marine natural products, which provide structurally distinct chemotypes capable of binding alternative tubulin sites and overcoming classical resistance pathways [40,41,71].

Recent reviews further confirm that marine-derived microtubule-targeting agents (MTAs) remain among the most promising sources of next-generation anticancer scaffolds, owing to their potency in multidrug-resistant cancer models and their ability to engage non-canonical tubulin binding domains [25,27,71].

#### 2.3.2. Marine Macrolides as Microtubule-Stabilizing Agents

Marine macrolides, predominantly polyketide-derived macrocyclic lactones, constitute a structurally diverse class of microtubule-stabilizing agents. In contrast to taxanes, many of these compounds bind to alternative sites on β-tubulin, thereby retaining activity in taxane-resistant tumor models [68,69,72].

Discodermolide promotes tubulin polymerization and suppresses microtubule dynamics, leading to G2/M arrest and apoptosis, and is notable for retaining activity in P-glycoprotein-overexpressing cells, indicating an ability to overcome efflux-mediated resistance [67,69,73]. In comparison, laulimalide, peloruside A, and zampanolide stabilize microtubules through binding to a distinct laulimalide/peloruside site on β-tubulin, separate from the taxane-binding pocket [69,70]. This alternative binding mode differentiates them from both taxanes and discodermolide, enabling reduced cross-resistance and potential synergistic combinations with taxane-based therapies.

Among these, peloruside A stands out for its strong in vivo efficacy and improved tolerability, with some models showing superior tumor inhibition compared to paclitaxel, likely due to its specific hydrogen-bonding interactions with β-tubulin that underpin its unique stabilization mechanism [68,69,72]. Overall, while all these macrolides converge on microtubule stabilization and mitotic arrest, they differ in tubulin-binding sites, resistance profiles, and therapeutic indices, collectively broadening the scope of tubulin-targeting anticancer strategies beyond classical taxanes.

#### 2.3.3. Marine Peptide-Based Microtubule Inhibitors

Marine-derived peptides involved in microtubule disruption exhibit a shared cytoskeletal target but clear distinctions in mechanism, clinical utility, and translational evolution. The dolastatin family, particularly dolastatin 10, represents the prototypical class, acting as a highly potent inhibitor of tubulin polymerization by binding near the vinca domain of β-tubulin, thereby preventing microtubule assembly and inducing mitotic arrest at nanomolar concentrations [19,27,39,74]. However, despite this potency, clinical application was limited by systemic toxicity and suboptimal pharmacokinetics, restricting its direct therapeutic use.

In contrast, synthetic derivatives such as monomethyl auristatin E (MMAE) exemplify a major translational advancement. While retaining the tubulin-inhibitory mechanism of dolastatin scaffolds, MMAE is primarily used as a payload in antibody–drug conjugates (ADCs), enabling targeted intracellular delivery to tumor cells. This approach significantly improves therapeutic index and reduces off-target toxicity, highlighting a shift from standalone cytotoxics to precision-guided delivery systems [25,39,75,76].

In the meantime, newly identified marine peptide-like compounds, often discovered through genome mining, display a broader and less defined interaction with the cytoskeleton. Although not all directly bind tubulin, they induce phenotypic effects consistent with microtubule disruption, such as mitotic arrest and spindle abnormalities [19,77]. Compared to dolastatin analogues, these compounds represent a mechanistically diverse and less optimized class, but they expand the chemical space and may reveal novel binding modes or indirect regulatory pathways affecting microtubule dynamics.

Overall, this comparison reveals a continuum from direct, high-affinity tubulin inhibitors with limited standalone use (dolastatin 10), to clinically successful, targeted derivatives (auristatin-based ADC payloads), and finally to emerging peptide scaffolds with broader and potentially novel mechanisms. This progression underscores how marine peptide natural products have evolved from potent cytotoxins into modular components of targeted cancer therapies, while still offering untapped potential for future microtubule-targeting agent development.

Concerning the newly identified peptides that induce phenotypic hallmarks of microtubule disruption, in vitro assays show they directly modulate tubulin polymerization, as measured by turbidimetry, fluorescence-based assays, and sedimentation of polymerized tubulin. Binding studies (SPR, ITC, photoaffinity labeling, and ligand competition with colchicine, vinblastine, or taxanes) confirm direct interaction with tubulin at functional binding sites [77].

In cells, these interactions produce classic mitotic defects, including G2/M arrest, spindle abnormalities, chromosome misalignment, and lagging chromosomes, as observed via immunofluorescence or live imaging. Live-cell analyses further reveal altered microtubule dynamics, including changes in growth and shrinkage rates, reduced stability, and disrupted EB1/EB3 plus-end behavior. Cells also exhibit either microtubule depolymerization or abnormal bundling, altered cold sensitivity, impaired nocodazole recovery, and shifts in tubulin acetylation and tyrosination, consistent with changes in microtubule stability [77].

Finally, these disruptions activate the spindle assembly checkpoint (e.g., MAD2, BUBR1), resulting in prolonged mitotic arrest and apoptosis. Structure–activity relationships further confirm specificity, as minor peptide modifications abolish tubulin binding and associated mitotic phenotypes [77].

#### 2.3.4. Mechanistic Diversity and Expansion of Tubulin Binding Sites

Marine microtubule-targeting natural products display mechanistic diversity through engagement of multiple distinct tubulin-binding sites, extending beyond classical taxane- and vinca-domain interactions. Macrolides such as discodermolide and peloruside A bind to a unique β-tubulin site separate from the taxane pocket, stabilizing microtubules through an alternative structural interface [67,69]. In contrast, dolastatin-derived analogues associate near the vinca domain, inhibiting tubulin polymerization but differing in binding kinetics and downstream cellular responses compared with classical vinca alkaloids.

Comparatively, these distinct binding modes translate into important functional differences. Taxane-site binders (discodermolide and peloruside A) act primarily as microtubule stabilizers, whereas vinca-site–proximal dolastatin analogues function as potent destabilizers, yet both converge on mitotic arrest and apoptotic induction. Importantly, this site-specific diversification reduces cross-resistance, enabling activity in tumors with β-tubulin mutations or efflux pump overexpression [68,69,70].

Furthermore, comparative pharmacological studies indicate that agents targeting non-overlapping tubulin sites may exhibit synergistic effects, as simultaneous disruption of multiple microtubule interfaces enhances mitotic catastrophe and apoptotic signaling [68,69,70]. Overall, marine-derived MTAs exemplify how structural diversity in tubulin binding translates into functional redundancy bypass, resistance circumvention, and potential therapeutic synergy.

#### 2.3.5. Challenges and Translational Perspectives

Despite their exceptional potency, marine-derived MTAs face substantial translational challenges, including limited natural abundance, structural complexity, and manufacturing constraints, particularly for large macrolides such as discodermolide and zampanolide [19,77,78]. Total synthesis strategies are often lengthy and low-yielding, thereby limiting clinical scalability.

However, advances in synthetic biology, semi-synthesis, and antibody–drug conjugate technology have partially mitigated these limitations. The successful clinical implementation of auristatin-based ADCs demonstrates a feasible strategy to overcome the toxicity and physicochemical limitations of peptide-based MTAs [25,78,79]. In parallel, ongoing structural biology efforts are enabling the rational design of simplified analogues that preserve tubulin-binding activity while improving drug-like properties.

#### 2.3.6. Conclusion, Remarks and Future Perspectives

**Figure 2:**
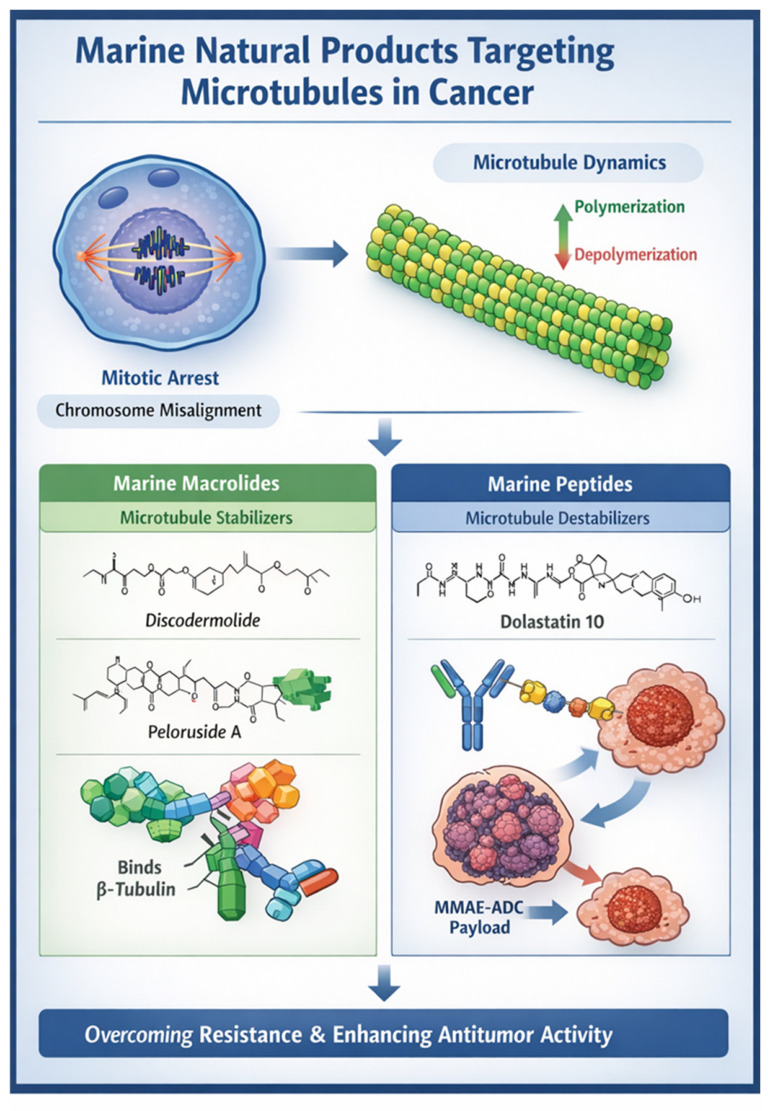
Mechanisms of action of representative marine natural products targeting microtubules in cancer.

Marine natural products, particularly macrolides and peptides, represent one of the most structurally diverse and mechanistically innovative sources of microtubule-targeting anticancer agents. Their ability to engage alternative tubulin binding sites overcomes drug resistance mechanisms and serves as scaffolds for antibody–drug conjugate development and underscores their continued relevance in contemporary oncology. Future progress will depend on the integration of synthetic biology, structural biology, and pharmacological optimization strategies to translate these complex marine-derived scaffolds into clinically viable therapeutics. In Figure 2, novel mechanisms of action of representative marine natural products targeting microtubule dynamics in cancer are illustrated.

### 2.4. Natural Products Targeting DNA and Transcriptional Machinery in Cancer

#### 2.4.1. DNA and Transcriptional Machinery as Anticancer Targets

DNA replication and transcriptional regulation represent fundamental vulnerabilities in rapidly proliferating cancer cells. In contrast to normal tissues, malignant cells exhibit elevated replication stress, epigenetic instability, and an increased dependence on transcriptional programs that sustain oncogene expression. Consequently, small molecules that directly bind DNA or disrupt transcriptional processes have historically yielded some of the most effective anticancer agents, including anthracyclines, bleomycins, and minor groove binders [6,79]. However, dose-limiting toxicities and the emergence of resistance have driven renewed interest in structurally novel natural products capable of selectively modulating DNA architecture and transcriptional machinery [1,10].

Recent advances in marine and microbial natural product discovery have further expanded this pharmacological class, revealing compounds that act not only through direct DNA damage but also via transcription factor inhibition, chromatin remodeling interference, and RNA polymerase blockade [42,43].

#### 2.4.2. DNA Minor Groove Binders and Transcriptional Disruption from Marine Sources

Marine-derived DNA- and transcription-targeting agents exhibit distinct yet complementary mechanisms of gene regulation interference, exemplified by trabectedin and bryostatins. Trabectedin (ET-743) acts as a direct DNA-binding agent, interacting with the minor groove at specific sequences, inducing helix distortion and selectively blocking transcription factor binding. This leads to targeted suppression of oncogenic transcription programs and promotes DNA strand breaks via transcription-coupled repair pathways, representing a precise, DNA-centric mechanism [80,81,82,83]. Ongoing analogue development aims to further refine sequence selectivity and reduce systemic toxicity [82,83,84].

In contrast, bryostatins function through an indirect, signaling-mediated mechanism, primarily as protein kinase C (PKC) modulators. Rather than binding DNA, they influence chromatin-associated signaling pathways, thereby altering transcriptional programs related to apoptosis and differentiation [85,86]. This positions bryostatins within a signal transduction-driven regulatory class, as opposed to the direct genomic interaction seen with trabectedin.

Comparatively, trabectedin represents a highly specific, DNA-targeting transcriptional inhibitor, whereas bryostatins exert broader, upstream modulation of gene expression via signaling networks. Together, these compounds illustrate two complementary strategies: direct structural interference with DNA (trabectedin) versus indirect transcriptional reprogramming through kinase signaling (bryostatins), highlighting the versatility of marine natural products in targeting cancer-associated transcriptional processes.

#### 2.4.3. DNA-Damaging and Strand-Cleaving Natural Products

Marine and microbial natural products that damage DNA exhibit extreme potency but mechanistic diversity in how they induce genomic instability, ranging from targeted chemical activation to broader oxidative damage pathways. Calicheamicin analogues represent the most potent and mechanistically precise class, binding to the DNA minor groove and undergoing reductive activation to generate diradical species, which induce site-selective double-strand breaks at picomolar concentrations [87]. This highly controlled “triggered cleavage” mechanism distinguishes calicheamicins as programmable DNA-cleaving agents, a property that has been directly translated into ADCs for targeted cancer therapy.

In contrast, marine-derived enediyne-like scaffolds and polyketide antibiotics exert DNA damage through a more diffuse, oxidative stress-mediated mechanism, generating ROS that cause replication fork collapse, single- and double-strand breaks, and secondary apoptotic signaling [88]. Compared with calicheamicins, these compounds are less sequence-specific but often induce broader genomic stress across multiple cellular compartments, reflecting a more generalized cytotoxic strategy.

Overall, calicheamicin analogues exemplify highly targeted, chemically activated DNA cleavage, whereas enediyne- and polyketide-derived agents represent ROS-driven, less site-specific DNA-damaging systems. Despite these mechanistic differences, both classes converge on irreversible genomic damage leading to apoptosis, underscoring DNA as a central but variably targeted vulnerability in marine and microbial anticancer natural products.

#### 2.4.4. Transcriptional Disruption via Epigenetic and Chromatin Modulation

Recent evidence indicates a shift in natural product anticancer mechanisms from direct DNA damage toward epigenetic and transcriptional regulation, with marine, fungal, and microbial metabolites targeting different layers of gene expression control. Marine-derived histone deacetylase (HDAC) inhibitors act primarily at the chromatin remodeling level, increasing histone acetylation, relaxing chromatin structure, and reactivating silenced tumor suppressor genes. This represents a reversible epigenetic reprogramming strategy, distinguishing these compounds from classical genotoxic agents by modulating accessibility rather than directly damaging DNA [89,90].

In contrast, fungal and microbial metabolites that inhibit RNA polymerase II or transcriptional elongation function at a downstream level, directly suppressing mRNA synthesis and global transcriptional output. These agents interfere with the core transcriptional machinery, leading to broad inhibition of oncogenic gene expression programs and rapid loss of survival signaling [90,91]. Compared to HDAC inhibitors, which primarily reshape chromatin structure, these compounds exert a more direct blockade of transcriptional execution.

Reasonably, both classes converge on transcriptional suppression and selective cancer cell apoptosis but differ in their molecular targets and regulatory hierarchy: HDAC inhibitors act upstream at the epigenetic/chromatin level, whereas RNA polymerase II inhibitors act downstream at the transcriptional machinery level. Together, these mechanisms reflect a broader conceptual transition in natural product pharmacology toward reprogramming or collapsing transcriptional dependency in cancer cells rather than inducing direct DNA lesions.

#### 2.4.5. Indirect Transcriptional Inhibition via the Signaling–DNA Axis

Recent evidence suggests that many contemporary natural products exert anticancer effects not through direct DNA interaction, but via indirect transcriptional regulation mediated by upstream signaling pathways that ultimately converge on chromatin and gene expression control. In this context, key compounds act by modulating oncogenic signaling hubs such as NF-κB, STAT3, and PI3K/Akt, leading to suppression of pro-survival gene transcription and activation of apoptotic programs [92,93].

Comparatively, marine alkaloids and chalcone derivatives predominantly operate through this kinase-dependent signaling mode, where inhibition of phosphorylation cascades upstream of the nucleus results in downstream transcriptional repression without direct DNA binding [93,94]. While both classes converge functionally on reduced tumor cell survival, marine alkaloids are often associated with broader pleiotropic signaling effects, whereas chalcones more frequently exhibit focused inhibition of specific kinase-driven pathways, such as PI3K/Akt or NF-κB axis modulation.

Taken together, these compounds contrast with classical DNA-intercalating agents by acting at an earlier regulatory level in the gene expression hierarchy. This signaling-to-transcription cascade mechanism is increasingly considered advantageous, as it enables effective suppression of oncogenic transcriptional networks while minimizing direct genotoxic stress and DNA damage-associated toxicity [93,94].

#### 2.4.6. Therapeutic Implications and Challenges

DNA- and transcription-targeting natural products remain among the most potent classes of anticancer agents; however, their clinical translation is limited by narrow therapeutic windows, off-target toxicity, and biosynthetic complexity. Trabectedin represents a successful example of clinical implementation, whereas most newly identified compounds remain at preclinical stages of development [95,96].

Current advances in targeted delivery systems (e.g., ADCs), synthetic biology, and genome-guided biosynthesis are increasingly addressing these limitations. In particular, conjugation of DNA-damaging natural products to tumor-specific antibodies has significantly improved selectivity, enabling the reconsideration of highly potent but previously clinically limited scaffolds [97,98].

#### 2.4.7. Conclusion, Remarks and Future Perspectives

Natural products targeting DNA integrity and transcriptional regulation remain a cornerstone of anticancer drug design and discovery. Recent advances indicate a clear expansion beyond classical DNA intercalators toward multifunctional agents that modulate transcription through direct DNA binding, epigenetic remodeling, and interference with oncogenic signaling pathways. Marine ecosystems and microbial biosynthetic systems continue to represent particularly rich sources of such compounds. Future progress will depend on the integration of structural biology, chemical biology, and precision delivery technologies to fully exploit their therapeutic potential while minimizing systemic toxicity. The key findings, representative compounds, and associated mechanisms of novel natural products targeting DNA and transcriptional machinery in cancer are summarized in Table 3.

### 2.5. Natural Products Targeting Signal Transduction Pathways in Cancer (PI3K/Akt, NF-κB, MAPK)

#### 2.5.1. Signal Transduction Pathways as Central Oncogenic Hubs

Aberrant activation of intracellular signaling networks is a hallmark of cancer progression, driving uncontrolled proliferation, resistance to apoptosis, angiogenesis, and metastatic dissemination. Among these, the PI3K/Akt/mTOR, NF-κB, and MAPK (Ras–Raf–MEK–ERK) pathways represent central regulatory hubs that integrate extracellular cues with transcriptional and metabolic outputs [100,101,102]. These pathways are frequently dysregulated in human malignancies through activating mutations in upstream receptors, loss of tumor suppressors (e.g., PTEN), and constitutive activation of oncogenic kinases, rendering them highly attractive therapeutic targets [103,104].

Natural products constitute a structurally diverse reservoir of modulators of these signaling cascades, offering multi-target regulatory activity, reduced toxicity, and chemical novelty compared with conventional synthetic inhibitors. Recent advances in metabolomics, phenotypic screening, and pathway-guided drug design and discovery have further expanded the identification of natural compounds capable of selectively interfering with oncogenic signal transduction [45,46,93,104].

#### 2.5.2. PI3K/Akt/mTOR Pathway Inhibition by Natural Products

The PI3K/Akt axis is a master regulator of cell survival, metabolism, and proliferation and is among the most frequently activated signaling pathways in cancer [46,100]. Recent studies indicate that natural products derived from flavonoids, alkaloids, terpenoids, and polyphenols exert potent inhibitory effects on this pathway [45,95].

Naturally derived compounds such as quercetin, berberine, curcumin, and ginsenosides converge mechanistically on the PI3K/Akt signaling axis, but differ in potency, pleiotropy, and downstream biological effects. Collectively, these agents inhibit PI3K phosphorylation and subsequent Akt activation, resulting in mTOR pathway suppression, disruption of cellular metabolic signaling, and induction of cell-cycle arrest and apoptosis [45,95].

Both quercetin and curcumin are broadly characterized as multi-target polyphenolic modulators, exerting additional effects on oxidative stress, inflammatory signaling, and kinase networks beyond PI3K/Akt, thereby contributing to their pleiotropic anticancer profiles [45,95]. Berberine, an isoquinoline alkaloid, more strongly emphasizes metabolic and mitochondrial regulation alongside PI3K/Akt inhibition, linking energetic stress to growth suppression. In contrast, ginsenosides and their derivatives are particularly associated with balanced modulation of PI3K/Akt-dependent autophagy and apoptosis, acting as key regulators of the transition between survival and programmed cell death under metabolic stress conditions [93,104].

Functionally, these compounds also share important higher-order effects, including reversal of chemoresistance and modulation of the tumor immune microenvironment, underscoring their role as both cytostatic and immunoregulatory agents [93,104]. Unlike classical small-molecule inhibitors, many of these natural products behave as partial pathway modulators rather than complete enzymatic blockers, which may contribute to a more favorable toxicity profile while preserving sufficient suppression of oncogenic signaling.

Overall, comparative analysis reveals a continuum of PI3K/Akt-targeting natural products ranging from highly pleiotropic phytochemicals (quercetin, curcumin) to metabolically focused alkaloids (berberine) and fine-tuned immunometabolic modulators (ginsenosides), collectively highlighting diverse strategies for modulating a central oncogenic signaling hub.

#### 2.5.3. NF-κB Signaling Inhibition and Inflammatory Reprogramming

The NF-κB pathway is a key regulator of inflammation-driven tumor progression, controlling the transcription of genes involved in cell survival, cytokine production, and metastasis [46,100]. Constitutive NF-κB activation is a frequent feature of multiple malignancies, including breast, colorectal, and hematological cancers [46,100].

Natural products including flavonoids (e.g., luteolin and apigenin), phenolic acids, and sesquiterpene lactones converge on a common anticancer mechanism involving suppression of the NF-κB signaling pathway, but differ in their chemical reactivity, target engagement, and broader biological effects. All three classes inhibit NF-κB activation primarily by blocking IκB kinase (IKK) phosphorylation, thereby preventing degradation of IκB and subsequent nuclear translocation of the p65 subunit, leading to transcriptional repression of anti-apoptotic genes (e.g., BCL-XL, XIAP) and pro-inflammatory cytokines such as TNF-α and IL-6 [45,100,102].

Comparatively, flavonoids such as luteolin and apigenin function as relatively broad-spectrum kinase modulators, exerting pleiotropic effects across multiple signaling pathways in addition to NF-κB, which contributes to their moderate potency but favorable safety profile. Phenolic acids, by contrast, generally exhibit weaker but more antioxidant-driven indirect inhibition of NF-κB signaling, often acting through modulation of oxidative stress-responsive upstream regulators. In contrast, sesquiterpene lactones typically display stronger and more direct NF-κB inhibition, often through covalent modification of cysteine residues in key signaling proteins, resulting in more potent but less selective biological effects [45,100,102].

Beyond intracellular signaling inhibition, these compounds also converge on higher-order tumor biology by contributing to tumor microenvironment remodeling, including suppression of pro-inflammatory macrophage polarization and inhibition of angiogenic signaling pathways, thereby reducing tumor-supportive inflammation and vascularization [93,103]. Collectively, comparative analysis highlights a continuum of NF-κB-targeting natural products ranging from multi-target regulatory flavonoids to redox-modulating phenolic acids to highly reactive electrophilic sesquiterpene lactones, all of which suppress tumor progression by linking inflammation control with apoptosis induction.

#### 2.5.4. MAPK/ERK Pathway Modulation by Natural Compounds

The MAPK signaling cascade (Ras–Raf–MEK–ERK) is critical for regulating cell proliferation, differentiation, and survival. Dysregulation of this pathway is highly prevalent in solid tumors, frequently driven by oncogenic mutations in Ras or BRAF [93,102].

Natural products that modulate MAPK signaling—primarily flavonoids, terpenoids, and alkaloids—exhibit a shared ability to suppress tumor proliferation, yet differ in their structural features, pathway specificity, and network-level effects. These compounds inhibit the MAPK cascade, most commonly by blocking ERK phosphorylation or upstream kinases such as MEK1/2, resulting in reduced transcription of key proliferation drivers including cyclin D1 and c-Myc [93,97].

Notably, flavonoids represent a broad class of polyphenolic signaling modulators that often exert moderate, pleiotropic inhibition across multiple kinase pathways, including MAPK, with relatively low toxicity. Terpenoids, by contrast, tend to display more structurally diverse and often stronger pathway-specific interactions, frequently targeting upstream kinases or membrane-associated signaling nodes that regulate MAPK activation. Alkaloids typically act through enzyme inhibition or receptor-mediated signaling interference, contributing to MAPK suppression alongside additional effects on apoptosis and cell-cycle control [45,97].

Beyond single-pathway modulation, compounds such as oridonin, honokiol, and resveratrol derivatives demonstrate dual inhibition of MAPK and NF-κB signaling, positioning them as network-level regulators rather than isolated pathway inhibitors. This dual targeting enhances their ability to overcome signaling redundancy and adaptive resistance mechanisms commonly observed in cancer cells [45,97].

Overall, comparative analysis highlights a continuum of MAPK-targeting natural products ranging from moderate, multi-target flavonoids to more structurally specialized terpenoids and alkaloids, culminating in integrated network modulators that simultaneously suppress MAPK and inflammatory NF-κB signaling, thereby amplifying anticancer efficacy through coordinated pathway disruption.

#### 2.5.5. Crosstalk Between PI3K/Akt, NF-κB, and MAPK Pathways

A defining feature of oncogenic signaling networks is extensive crosstalk among the PI3K/Akt, NF-κB, and MAPK pathways. Activation of one pathway often compensates for inhibition of another, thereby promoting therapeutic resistance [93,101]. In contrast, natural products frequently exert simultaneous modulation across multiple pathways, which may account for their robust anticancer activity in preclinical models [93,101].

Recent integrative analyses indicate that numerous phytochemicals and microbial metabolites concurrently inhibit PI3K/Akt and NF-κB signaling while suppressing MAPK activity, thereby inducing apoptosis through combined mitochondrial and transcriptional mechanisms [44,45,104]. This system-level regulatory activity represents a major advantage over highly selective kinase inhibitors.

#### 2.5.6. Therapeutic Implications and Future Perspectives

Despite strong preclinical evidence, clinical translation of natural product-derived signaling inhibitors remains limited by poor oral bioavailability, metabolic instability, and potential off-target effects. However, recent advances in nano-delivery systems, prodrug strategies, and semi-synthetic optimization are significantly improving pharmacokinetic properties and clinical feasibility [45,46].

Future research directions are increasingly focused on: (i) artificial intelligence-assisted identification of signaling inhibitors, (ii) multi-omics-guided pathway mapping, (iii) rational combination strategies with immunotherapy and kinase inhibitors, and (iv) structural optimization of multi-target natural scaffolds. These approaches are expected to accelerate the development of next-generation anticancer therapeutics derived from natural sources [101,104].

#### 2.5.7. Conclusion Remarks

Natural products targeting PI3K/Akt, NF-κB, and MAPK signaling pathways represent a highly promising class of anticancer agents with multifunctional regulatory properties. Recent evidence demonstrates that these compounds act not as simple single-target inhibitors but rather as systems-level modulators of oncogenic signaling networks, enabling simultaneous control of proliferation, survival, inflammation, and apoptosis. Continued integration of molecular pharmacology with advanced drug design and discovery technologies will be essential for translating these compounds into clinically effective anticancer therapies. The key findings, representative naturally derived compounds, and mechanisms of natural products targeting oncogenic signal transduction pathways are summarized in Table 4.

### 2.6. Natural Products as Epigenetic Modulators in Cancer Therapy

#### 2.6.1. Epigenetic as a Reversible Cancer Vulnerability

Epigenetic regulation comprises heritable and reversible modifications in gene expression that occur without alterations to the underlying DNA sequence. These mechanisms include DNA methylation, histone post-translational modifications (e.g., acetylation, methylation, and phosphorylation), chromatin remodeling, and non-coding RNA-mediated regulation [105,106,107]. In cancer, epigenetic dysregulation contributes to the silencing of tumor suppressor genes, activation of oncogenes, and global chromatin instability, thereby promoting tumor initiation and progression [105,106,107].

In contrast to genetic mutations, epigenetic alterations are, in principle, pharmacologically reversible, rendering key epigenetic regulators—such as DNA methyltransferases (DNMTs), HDACs, and histone methyltransferases (HMTs)—attractive therapeutic targets [105,106,108]. In this context, natural products represent a structurally and functionally diverse reservoir of epigenetic modulators capable of reprogramming aberrant transcriptional states in cancer cells [105,106,108].

Recent studies further expand this landscape by demonstrating that natural compounds modulate not only canonical targets such as DNMTs and HDACs but also more complex epigenetic regulators, including EZH2 and LSD1, thereby broadening the druggable epigenetic space [48,49,90,109].

#### 2.6.2. DNA Methylation Modulation by Natural Products

DNA methylation, primarily catalyzed by DNMT1, DNMT3A, and DNMT3B, plays a central role in transcriptional silencing in cancer. DNMT hyperactivity leads to promoter hypermethylation of tumor suppressor genes, whereas global hypomethylation contributes to genomic instability [108,109,110].

Natural products such as epigallocatechin gallate (EGCG), curcumin, genistein, and resveratrol function as weak to moderate DNMT inhibitors, but differ in their dominant mechanisms of action, target engagement, and epigenetic breadth. These compounds inhibit DNMT activity either through direct enzyme binding or through transcriptional downregulation of DNMT expression, leading to reactivation of silenced tumor suppressor genes such as p16INK4a and BRCA1, and subsequent induction of cell-cycle arrest and apoptosis [47,110].

Comparatively, EGCG (a green tea catechin) is among the most well-characterized DNMT-interacting polyphenols, with evidence supporting direct binding to DNMT catalytic sites, giving it relatively stronger enzymatic inhibition compared with other dietary polyphenols [47,110]. Curcumin, in contrast, acts more prominently through transcriptional repression of DNMTs and broad epigenetic modulation, including histone modification and chromatin remodeling. Genistein, an isoflavone, exhibits a more pathway-integrated epigenetic profile, combining DNMT suppression with estrogen receptor-mediated signaling effects, while resveratrol functions as a multi-target epigenetic regulator, influencing DNMT activity indirectly alongside sirtuin activation and broader metabolic effects [47,110].

Beyond direct or transcriptional DNMT inhibition, these polyphenols also interfere with the S-adenosylmethionine (SAM)-dependent methyl donor pathway, thereby reducing intracellular methylation capacity and amplifying global hypomethylation effects in cancer cells [48,49]. Compared to enzymatic inhibition alone, this dual mechanism—DNMT suppression plus metabolic interference—enhances epigenetic reprogramming potential and distinguishes natural compounds from many synthetic DNMT inhibitors that act primarily through single-target enzymatic blockade [48,49].

Conclusively, comparative analysis reveals a continuum of epigenetic modulation: EGCG as a relatively direct DNMT inhibitor, curcumin and resveratrol as broad epigenetic and metabolic regulators, and genistein as a signaling-integrated epigenetic modulator, together illustrating the multifunctional nature of natural products in cancer epigenetic therapy.

#### 2.6.3. Histone Deacetylase (HDAC) Inhibition and Chromatin Relaxation

Histone acetylation is a dynamic epigenetic modification regulated by histone acetyltransferases (HATs) and HDACs. HDAC overexpression is frequently observed in cancer and is associated with transcriptional repression of pro-apoptotic and tumor suppressor genes [110,111].

Naturally derived HDAC inhibitors—including butyrate, sulforaphane, curcumin, and apigenin—form a mechanistically related but functionally diverse class of epigenetic modulators that promote chromatin relaxation and reactivation of tumor suppressor gene programs through increased histone acetylation [49,90,111]. Despite converging on HDAC inhibition, these compounds differ substantially in chemical class, potency, selectivity, and breadth of downstream epigenetic effects.

Butyrate, a short-chain fatty acid produced by gut microbiota, acts as a broad-spectrum, reversible HDAC inhibitor, often affecting multiple HDAC isoforms and serving as a key link between metabolic state and epigenetic regulation. Sulforaphane, an isothiocyanate from cruciferous vegetables, demonstrates a more selective HDAC inhibitory profile, particularly in prostate and breast cancer models, and induces strong increases in histone H3 and H4 acetylation, thereby activating apoptosis-associated transcriptional programs [49,90]. In contrast, curcumin exhibits weaker direct HDAC inhibition but broader epigenetic modulation, functioning alongside effects on transcription factors and inflammatory signaling, while apigenin represents a more moderate HDAC modulator with pleiotropic kinase and gene-regulatory effects, contributing to combined epigenetic and signaling-level control [49,90].

Importantly, many of these natural HDAC inhibitors display isoform selectivity or partial inhibitory activity, which distinguishes them from classical synthetic HDAC inhibitors that often act as potent pan-inhibitors. This partial and context-dependent inhibition is increasingly considered advantageous, as it may preserve anticancer efficacy while reducing systemic toxicity and off-target epigenetic disruption [110,111].

Overall, comparative analysis highlights a spectrum of natural HDAC modulation ranging from metabolite-derived broad inhibitors (butyrate) to selective phytochemical inhibitors (sulforaphane) and multi-target epigenetic regulators (curcumin and apigenin), collectively underscoring the structural and functional diversity of dietary and plant-derived epigenetic therapeutics.

#### 2.6.4. Histone Methylation and Demethylation Targeting

An emerging area in epigenetic drug discovery involves the modulation of histone methylation states via enzymes such as EZH2 (H3K27 methyltransferase) and LSD1 (lysine-specific demethylase 1), which regulate chromatin compaction and transcriptional repression in cancer [110,111].

Flavonoids, curcuminoids, and stilbene derivatives represent a functionally convergent group of natural products that modulate histone methylation landscapes, yet differ in their dominant molecular targets, breadth of epigenetic activity, and regulatory intensity. Collectively, these compounds restore the expression of silenced tumor suppressor genes by suppressing EZH2 (a histone methyltransferase “writer”) and inhibiting LSD1 (a histone demethylase “eraser”), thereby reversing repressive chromatin states associated with oncogenesis [47,48,90].

Comparatively, flavonoids act as relatively broad epigenetic modulators with pleiotropic effects on both chromatin-associated enzymes and upstream signaling pathways, resulting in moderate but wide-ranging transcriptional reactivation. Curcuminoids, particularly curcumin and its analogues, exhibit more pronounced multi-target epigenetic activity, simultaneously affecting histone methylation, acetylation, and transcription factor signaling, which positions them as central “network-level” regulators of chromatin dynamics. In contrast, stilbene derivatives (e.g., resveratrol-related scaffolds) tend to exert more selective regulatory effects, often influencing specific epigenetic enzymes indirectly through metabolic and stress-response pathways rather than strong direct enzymatic inhibition [47,48,90].

Critically, across all three classes, histone methylation modulation is not restricted to single enzyme inhibition but instead involves simultaneous regulation of both “writer” (e.g., EZH2) and “eraser” (e.g., LSD1) enzymes, resulting in more extensive chromatin remodeling compared with conventional single-target epigenetic drugs. This multi-enzyme regulatory behavior is particularly relevant in epigenetically driven malignancies, where transcriptional silencing rather than genetic mutation predominates as the key oncogenic mechanism [47,48,90].

Hence, comparative analysis highlights a continuum of histone methylation modulators ranging from moderately broad flavonoids to highly pleiotropic curcuminoids and more selectively acting stilbene derivatives, collectively emphasizing the capacity of natural products to reprogram chromatin states through coordinated epigenetic enzyme regulation.

#### 2.6.5. Non-Coding RNA and Chromatin-Associated Regulation

Beyond classical epigenetic enzymes, increasing evidence indicates that natural products also regulate microRNAs (miRNAs) and long non-coding RNAs (lncRNAs), which play critical roles in post-transcriptional gene regulation [47,48].

Both resveratrol and curcumin represent two well-studied polyphenols that modulate cancer biology through miRNA-mediated regulatory mechanisms, extending epigenetic control beyond DNA and histone modifications into post-transcriptional RNA networks. Both compounds influence oncogenic and tumor-suppressive miRNAs, including downregulation of miR-21 (a key oncomiR) and restoration of tumor-suppressive miRNA expression, thereby indirectly reshaping gene expression programs involved in proliferation, apoptosis, and survival signaling [47,48].

Resveratrol primarily acts as a broad metabolic and signaling modulator, influencing miRNA networks indirectly through pathways such as sirtuin activation, oxidative stress regulation, and inflammatory signaling. This positions it as a more system-level regulator of RNA-mediated epigenetic circuits. In contrast, curcumin exerts a more pleiotropic regulatory effect, directly impacting multiple transcription factors (e.g., NF-κB, STAT3) alongside miRNA modulation, resulting in stronger integration of epigenetic, transcriptional, and post-transcriptional control layers [47,48].

Together, these compounds illustrate a shared but hierarchically distinct ability to regulate RNA-based epigenetic circuitry, where resveratrol emphasizes metabolic–epigenetic coupling, while curcumin provides broader multi-level regulatory suppression of oncogenic networks. Collectively, this expands the concept of epigenetic modulation to include miRNA-centered regulatory axes, reinforcing the therapeutic versatility of polyphenolic natural products.

#### 2.6.6. Systems-Level Epigenetic Reprogramming by Natural Products

An emerging paradigm is that natural products rarely act through a single epigenetic target. Instead, they function as system-level epigenetic modulators that simultaneously influence DNA methylation, histone modifications, and chromatin accessibility [47,48,90].

Both curcumin and quercetin represent two pleiotropic polyphenols that induce coordinated epigenetic reprogramming, but they differ in their relative emphasis on specific regulatory layers and breadth of molecular interactions. Both compounds converge on a multi-level epigenetic signature involving DNMT downregulation, HDAC inhibition, increased histone acetylation, and miRNA network modulation, ultimately leading to reactivation of tumor suppressor genes and suppression of oncogenic signaling pathways [47,48,90].

Curcumin functions as a highly network-oriented epigenetic modulator, with strong effects on transcription factor signaling (e.g., NF-κB, STAT3) that amplify its downstream impact on DNA methylation, histone modification, and miRNA expression. This positions curcumin as a broad epigenetic “hub regulator” capable of simultaneously influencing multiple layers of gene control. In contrast, quercetin tends to act as a more balanced multi-target modulator, with relatively stronger emphasis on enzyme-level regulation (DNMTs and HDACs) and antioxidant-mediated signaling effects that indirectly shape chromatin accessibility and miRNA profiles [47,48,90].

Functionally, both compounds achieve similar biological outcomes—namely reactivation of tumor suppressor pathways and inhibition of oncogenic transcriptional programs—but through slightly different emphases: curcumin integrates more strongly into transcriptional signaling networks, whereas quercetin more directly modulates epigenetic enzymes and oxidative stress-linked regulatory pathways. Together, they exemplify how natural products can orchestrate multi-layered epigenetic reprogramming, reinforcing the concept of systems-level cancer regulation rather than single-target inhibition.

#### 2.6.7. Therapeutic Challenges and Future Directions

Despite strong preclinical evidence, the clinical translation of epigenetic natural products remains limited by low bioavailability, metabolic instability, and suboptimal pharmacokinetic properties. In addition, many compounds exhibit moderate potency compared with synthetic epigenetic drugs.

Nevertheless, recent advances in nanocarrier-based delivery systems, structural optimization, and combination therapy strategies are significantly improving their therapeutic potential. Future research is increasingly focused on: (i) AI-guided epigenetic target prediction, (ii) synthetic optimization of natural scaffolds, (iii) combination strategies with immunotherapy, and (iv) multi-omics-driven epigenome mapping. These approaches are expected to enhance both the selectivity and clinical applicability of natural epigenetic modulators.

#### 2.6.8. Conclusions

**Figure 3:**
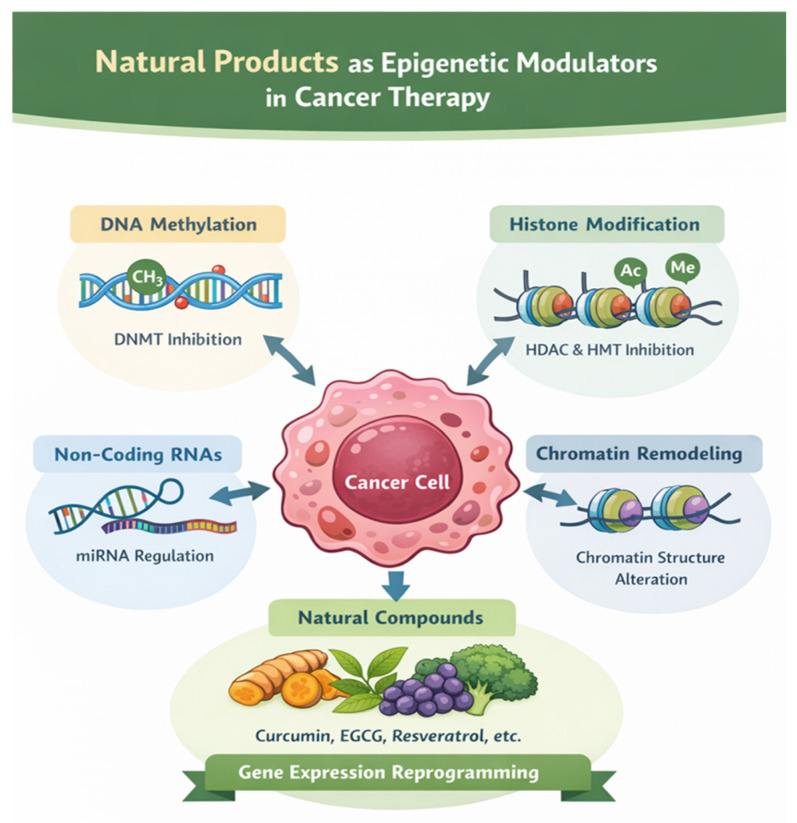
Key mechanisms of natural products as epigenetic modulators in cancer.

Natural products constitute a highly diverse and mechanistically versatile class of epigenetic modulators in cancer therapy. Recent advances demonstrate that these compounds act across multiple layers of epigenetic regulation, including DNA methylation, histone acetylation, histone methylation, and non-coding RNA networks. Their ability to reprogram aberrant transcriptional landscapes positions them as promising candidates for next-generation anticancer strategies, particularly when integrated with modern drug delivery platforms and systems biology approaches. The key findings, representative compounds, and mechanisms of natural products acting as epigenetic modulators in cancer are summarized in Figure 3.

### 2.7. Natural Products Targeting Immune Modulation and the Tumor Microenvironment in Cancer Therapy

#### 2.7.1. The Tumor Microenvironment (TME) as an Immunological Regulatory Hub

Cancer progression is now widely recognized as not solely driven by tumor-cell-intrinsic alterations but as the result of dynamic interactions between malignant cells and a complex tumor microenvironment (TME). The TME comprises immune cells, cancer-associated fibroblasts, endothelial cells, extracellular matrix components, and a broad range of soluble mediators [112,113]. Within this ecosystem, tumor cells actively reshape immune surveillance by promoting immunosuppressive cell populations, including regulatory T cells (Tregs), tumor-associated macrophages (TAMs), and myeloid-derived suppressor cells (MDSCs), while concurrently suppressing cytotoxic CD8^+^ T cell activity [112,113].

This immunosuppressive reprogramming represents a central mechanism of immune evasion and therapeutic resistance. Accordingly, targeting the TME has emerged as a cornerstone of contemporary cancer immunotherapy, particularly through strategies aimed at restoring effector immune function and reversing suppressive signaling networks [50,113].

In this context, natural products have attracted increasing interest due to their pleiotropic immunomodulatory properties, capacity to regulate cytokine networks, and ability to reprogram immune cell phenotypes, often with comparatively low systemic toxicity [114].

#### 2.7.2. Natural Products Targeting Innate Immune Suppression in the TME

A key immunosuppressive feature of the TME is the accumulation of M2-polarized TAMs and MDSCs, which inhibit T cell activation through secretion of immunosuppressive cytokines such as IL-10 and TGF-β, as well as through arginase-mediated depletion of L-arginine.

Natural products including polysaccharides, flavonoids, alkaloids, and triterpenoids converge on tumor immunomodulation, but differ substantially in their primary immune targets, signaling specificity, and depth of reprogramming within the tumor microenvironment. In fact, polysaccharides, particularly those derived from plants, primarily act as broad immunostimulatory agents, promoting macrophage reprogramming from the pro-tumoral M2 phenotype to the anti-tumoral M1 state. This shift enhances antigen presentation, cytokine production, and CD8^+^ T-cell-mediated cytotoxicity, reflecting a predominantly innate immune-driven mechanism with systemic immune-activating properties [50,51].

In contrast, flavonoids exert more signal-targeted immunomodulatory effects, often influencing macrophage polarization indirectly through suppression of NF-κB- and STAT3-driven inflammatory signaling. This results in reduced pro-tumoral cytokine expression and partial reversal of immunosuppressive signaling, positioning flavonoids as moderate, multi-pathway regulators of immune tone rather than strong immune activators [50,51].

Alkaloids, while less uniformly characterized in this context, typically display pleiotropic immunoregulatory activity, affecting both macrophage polarization and broader immune signaling networks. Their effects often combine direct modulation of intracellular signaling pathways with secondary anti-inflammatory outcomes, leading to context-dependent shifts in immune cell function. Finally, triterpenoids stand out as relatively strong suppressors of immunosuppressive myeloid populations, particularly MDSCs. They inhibit key regulatory pathways such as STAT3 and NF-κB, thereby blocking MDSCs’ expansion and function and indirectly enhancing CD8^+^ T-cell infiltration and antitumor activity [114]. Compared with polysaccharides, triterpenoids act more specifically on pathological immune suppression circuits rather than broadly stimulating innate immunity.

Overall, comparative analysis reveals a functional spectrum of immune reprogramming: polysaccharides act as broad innate immune activators, flavonoids and alkaloids function as signaling modulators that recalibrate inflammatory pathways, and triterpenoids serve as targeted inhibitors of immunosuppressive myeloid networks, collectively converging on enhanced antitumor immunity through complementary mechanisms.

#### 2.7.3. Modulation of Adaptive Immunity: T Cells and Immune Checkpoint Regulation

Adaptive immune suppression within the TME is primarily driven by dysfunctional CD8^+^ T cells, expansion of Tregs, and upregulation of immune checkpoint pathways such as PD-1/PD-L1. Natural products have demonstrated considerable potential to restore T cell effector function through multiple complementary mechanisms [112,113].

Polyphenols and flavonoids act as T cell-oriented immunomodulators, but differ in the breadth of immune effects, signaling intensity, and degree of synergy with checkpoint blockade. Collectively, these compounds enhance T cell receptor (TCR) signaling, increase production of key effector cytokines such as IL-2 and IFN-γ, and strengthen cytotoxic T lymphocyte (CTL) function, thereby improving antitumor immune surveillance [112,113].

Notably, polyphenols such as rosmarinic acid demonstrate a more integrated immunometabolic reprogramming profile, simultaneously enhancing T cell activation and shifting cellular metabolism toward states that support sustained effector function. This results in increased tumor infiltration by activated T cells and improved in vivo antitumor responses [52,113]. In contrast, flavonoids generally act as more broad signaling modulators, enhancing TCR-associated signaling cascades and cytokine production while also exerting auxiliary anti-inflammatory effects that can indirectly shape the immune microenvironment [52,113].

Beyond direct T cell activation, both classes contribute to immune checkpoint sensitization, but through complementary mechanisms. They reduce immunosuppressive cytokine production and downregulate PD-L1 expression on tumor and stromal cells, thereby improving responsiveness to anti-PD-1/PD-L1 therapies [112,113]. However, polyphenols tend to exert stronger effects on metabolic and transcriptional reprogramming of T cells, whereas flavonoids are more associated with the modulation of upstream signaling networks that govern immune activation thresholds.

Collectively, comparative analysis highlights a functional continuum in which flavonoids primarily tune immune signaling intensity, while specific polyphenols such as rosmarinic acid additionally rewire T cell metabolism and effector persistence, together enhancing both intrinsic T cell function and extrinsic checkpoint therapy efficacy.

#### 2.7.4. Cytokine Networks and Inflammatory Reprogramming in the TME

A defining feature of tumor-promoting inflammation is the dysregulation of cytokine signaling, characterized by elevated levels of IL-6, TNF-α, and IL-10, which collectively sustain tumor progression and immune suppression. Natural products have been extensively reported to modulate these cytokine networks [114,115,116].

Flavonoids, terpenoids, and alkaloids converge on TME reprogramming, but differ in their dominant immunological targets, signaling breadth, and depth of cytokine modulation. Collectively, these natural product classes regulate key inflammatory mediators—including IL-1β, IL-6, IL-12, and TNF-α—thereby shifting the TME from a pro-tumoral inflammatory state toward an immune-activating environment [51,114].

In general, flavonoids function as broad-spectrum anti-inflammatory modulators, exerting balanced suppression of multiple cytokines through regulation of upstream signaling pathways such as NF-κB and MAPK [114,115,116]. This results in a gradual rebalancing of immune tone rather than highly selective pathway targeting. Terpenoids, by contrast, often display more potent and pathway-focused immunomodulatory effects, strongly influencing NF-κB-driven cytokine production and thereby producing more pronounced shifts in inflammatory signaling networks within the TME. Alkaloids tend to exhibit context-dependent and pleiotropic immunoregulatory activity, modulating both pro- and anti-inflammatory cytokines through interactions with multiple signaling axes, including STAT- and kinase-mediated pathways [114,115,116].

Functionally, these cytokine changes are not isolated events but are tightly linked to downstream immune activation. Across all three classes, suppression of pro-inflammatory cytokines contributes to restoration of dendritic cell maturation and improved antigen presentation, both of which are essential for initiating effective antitumor T cell responses [115,116]. However, flavonoids tend to support a more balanced immune normalization, terpenoids often drive a stronger inflammatory reset, and alkaloids provide a broader but less predictable immunomodulatory profile.

Taken together, comparative analysis highlights a continuum of TME reprogramming strategies: flavonoids as broad immune balancers, terpenoids as stronger cytokine suppressors, and alkaloids as multifunctional modulators, all converging on improved antigen presentation and enhanced antitumor immunity.

#### 2.7.5. Remodeling of the TME and Immune Cell Infiltration

One of the most significant outcomes of natural product-based intervention is structural and functional remodeling of the TME, including increased infiltration of cytotoxic lymphocytes and reduced stromal-mediated immune exclusion [113,114].

Recent reviews highlight that natural products can: (a) reduce extracellular matrix stiffness and tumor fibrosis, (b) inhibit angiogenesis through VEGF suppression, (c) enhance CD8^+^ T cell infiltration into tumor tissue, and (d) suppress Treg recruitment and differentiation. Collectively, these effects contribute to the conversion of “immune-cold” tumors into “immune-hot” tumors, thereby improving responsiveness to immunotherapy [113,114].

#### 2.7.6. Systems-Level Immune Modulation: Multi-Target Advantages of Natural Products

A major advantage of natural products is their ability to function as systems-level immune regulators rather than single-target agents. In contrast to monoclonal antibodies or selective kinase inhibitors, many natural compounds simultaneously modulate multiple components of the immune–tumor interface, including: (a) macrophage polarization, (b) T cell activation, (c) cytokine secretion, (d) immune checkpoint expression, and (e) stromal signaling pathways [51,112,113,114].

This multi-layered regulatory capacity is particularly relevant in the TME, where extensive pathway redundancy often compensates for single-node inhibition, thereby limiting the efficacy of highly specific therapeutic agents [51,112,113,114].

#### 2.7.7. Challenges and Future Perspectives

Despite robust preclinical evidence, the clinical translation of immunomodulatory natural products remains limited by several factors, including (a) low bioavailability and metabolic instability, (b) insufficient tumor-specific accumulation, (c) variability in biological potency, and (d) limited clinical validation.

Emerging strategies such as nanoparticle-based delivery systems, structural optimization of lead compounds, and rational combination immunotherapy approaches are actively addressing these limitations. In particular, the combination of natural products with immune checkpoint inhibitors represents a promising strategy to enhance therapeutic efficacy and overcome resistance mechanisms [113,114].

#### 2.7.8. Conclusion Remarks

Natural products constitute a highly versatile and mechanistically diverse class of immunomodulatory agents capable of reshaping the TME. Recent advances demonstrate that these compounds not only regulate immune cell populations but also reprogram cytokine networks and restore anti-tumor immunity at a systems level. Their capacity to convert immunosuppressive TMEs into immunologically active environments positions them as promising adjuncts to modern cancer immunotherapy, particularly in combination with immune checkpoint blockade strategies. In Table 5, the key immunological findings, mechanisms of action, and immune targets affected by natural products in cancer therapy are reported.

### 2.8. Translational Limitations and the In Vitro-to-Clinical Gap in Natural Anticancer Therapeutics over the Last Five Years

Despite a rapidly expanding body of preclinical evidence demonstrating broad anticancer activities of natural products across multiple biological hallmarks of cancer, a persistent and well-documented translational gap remains between in vitro and in vivo efficacy and clinically effective therapeutics. Over the last five years, this gap has become increasingly apparent as compounds such as curcumin, resveratrol, EGCG, sulforaphane, and genistein continue to show multifaceted anticancer actions—including induction of apoptosis, inhibition of proliferation, suppression of angiogenesis, modulation of immune responses, reduction in oxidative stress, and attenuation of invasion and metastasis—in cell-based and animal models, yet fail to demonstrate consistent clinical benefit in controlled human trials [117,118]. This disconnect reflects a combination of pharmacokinetic constraints, tumor biological complexity, and methodological limitations in preclinical study design.

A primary limitation is poor systemic bioavailability, which remains the dominant barrier for most polyphenolic and phytochemical anticancer agents. Many compounds exhibit low aqueous solubility, rapid hepatic metabolism, and extensive first-pass glucuronidation or sulfation, resulting in plasma concentrations far below those required to reproduce the antiproliferative, pro-apoptotic, or anti-metastatic effects observed in vitro [119,120]. For example, curcumin and resveratrol consistently demonstrate modulation of apoptosis-related pathways (e.g., caspase activation, Bcl-2 family regulation), cell cycle arrest, and inhibition of NF-κB and PI3K/Akt signaling at micromolar concentrations, whereas clinical studies typically achieve nanomolar systemic exposure, raising fundamental questions about physiological relevance [119,120]. Even with advanced delivery strategies such as nanoparticles, liposomes, and phospholipid complexes, achieving sustained tumor-site accumulation and predictable pharmacological exposure remains inconsistent [119,120].

A second major issue is over-reliance on reductionist in vitro systems, which fail to capture the full complexity of tumor biology. Cancer progression is driven not only by intrinsic tumor cell signaling but also by dynamic interactions within the tumor microenvironment, including hypoxia, immune infiltration, stromal remodeling, metabolic competition, and extracellular matrix signaling [121]. Two-dimensional monocultures do not adequately reproduce these conditions, leading to overestimation of compound efficacy in processes such as apoptosis induction, anti-angiogenic signaling, and metastasis suppression [122]. Consequently, mechanistic findings observed in simplified systems often fail to translate into durable tumor regression in vivo, where compensatory pathways and cellular heterogeneity rapidly restore malignant phenotypes.

In addition, many natural anticancer agents act as multi-target, low-affinity modulators, which complicates translational predictability. While pleiotropic activity is often considered advantageous in oncology due to pathway redundancy, it introduces challenges in dose standardization, off-target effect characterization, and regulatory evaluation [1,2]. Unlike conventional targeted therapies designed for high specificity, these compounds frequently influence multiple signaling networks simultaneously, including inflammatory pathways (e.g., NF-κB, COX-2), survival signaling (e.g., MAPK, PI3K/Akt), angiogenic regulators (e.g., VEGF), and apoptotic machinery [1,2]. This broad activity spectrum complicates the identification of robust pharmacodynamic biomarkers and limits the ability to define patient subgroups most likely to benefit.

Another critical limitation is the lack of standardized clinical endpoints and validated translational biomarkers [123]. Although preclinical studies frequently report changes consistent with anticancer activity—such as increased apoptotic index, reduced tumor volume in xenografts, decreased angiogenic markers, or modulation of epithelial–mesenchymal transition (EMT) markers—these endpoints are rarely validated in human tumor tissues or correlated with long-term clinical outcomes [123]. Inter-patient variability in metabolism, microbiome composition, immune status, and tumor genetic background further complicates reproducibility and contributes to inconsistent trial results.

Finally, heterogeneity in preclinical study design and publication bias continue to inflate perceived therapeutic potential [124]. Many studies rely on supra-physiological dosing, short exposure periods, and selective reporting of positive findings, which collectively overstate translational promise [124]. This is particularly evident in natural product oncology research, where mechanistic breadth is often not matched by rigorous pharmacological validation, clinically relevant dosing regimens, or standardized delivery approaches [124].

Collectively, these limitations highlight a fundamental disconnect between broad anticancer mechanisms observed in simplified experimental systems and the complex pharmacological and biological realities of human cancer. Bridging this gap will require integration of physiologically relevant models (3D organoids, patient-derived xenografts, and immune-competent systems), improved drug delivery technologies, quantitative pharmacokinetic–pharmacodynamic modeling, and biomarker-driven clinical trial designs. Without such integration, many natural anticancer compounds are likely to remain mechanistically compelling but clinically underexploited candidates in oncology.

### 2.9. Previous Clinically Validated Natural Product-Derived Anticancer Agents

A limited but highly significant group of previous naturally derived compounds has achieved robust clinical validation and remains central to modern oncology practice. Unlike most novel phytochemicals currently investigated in preclinical settings, these agents have undergone extensive pharmacological optimization, clinical trials, and regulatory approval, establishing them as standard-of-care chemotherapeutics. The most prominent classes include taxanes, vinca alkaloids, and camptothecin derivatives, all of which originate from plant secondary metabolites but are now primarily used in semi-synthetic or optimized clinical forms.

Taxanes, including paclitaxel, docetaxel, and cabazitaxel, are derived from Taxus species and exert potent anticancer effects by stabilizing microtubules and suppressing their dynamic instability, thereby inducing mitotic arrest at the G2/M phase and triggering apoptosis in rapidly proliferating tumor cells. Clinically, paclitaxel is widely used in ovarian, breast, lung, and pancreatic cancers, while docetaxel is approved for breast, prostate, gastric, and head-and-neck malignancies [125,126]. Cabazitaxel, a second-generation taxane, was developed to overcome docetaxel resistance and is clinically used in metastatic castration-resistant prostate cancer, demonstrating improved efficacy in taxane-resistant tumor populations.

The vinca alkaloids, including vincristine and vinblastine, are derived from Catharanthus roseus and function as microtubule-destabilizing agents. They bind to β-tubulin and inhibit microtubule polymerization, leading to metaphase arrest and disruption of mitotic spindle formation. Vincristine is primarily used in hematological malignancies such as acute lymphoblastic leukemia and lymphomas [127], whereas vinblastine is used in Hodgkin lymphoma, testicular cancer, and other solid tumors [128]. These agents remain essential components of combination chemotherapy regimens due to their strong cytotoxic activity and synergistic effects with other anticancer drugs.

Camptothecin-derived agents, including irinotecan and topotecan, originate from Camptotheca acuminata and target DNA topoisomerase I, thereby stabilizing the cleavable enzyme–DNA complex and inducing irreversible DNA strand breaks. Irinotecan is clinically approved for colorectal cancer and is widely used in combination regimens such as FOLFIRI [129], while topotecan is indicated for ovarian cancer, small-cell lung cancer, and cervical cancer [130]. These agents represent a cornerstone of DNA-damage-based chemotherapy and remain critical in both first-line and second-line treatment protocols.

Collectively, these clinically validated natural product-derived agents demonstrate that plant-derived compounds can be successfully translated into effective anticancer therapies when supported by rigorous pharmacological optimization, structural modification, and clinical validation. However, their success contrasts sharply with the majority of natural compounds currently investigated in preclinical oncology, which often lack comparable pharmacokinetic optimization, dosing standardization, and clinical efficacy evidence.

## 3. Discussion

**Figure 4:**
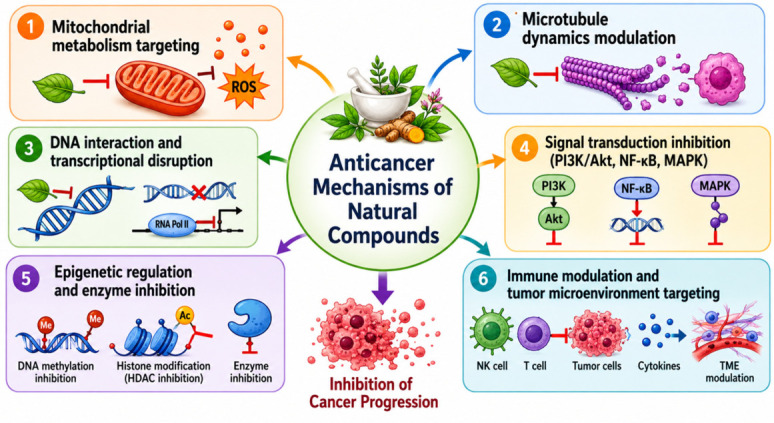
The well-known anticancer mechanisms of action of natural compounds.

The expanding landscape of natural products in anticancer drug discovery reflects a substantive conceptual transition from the classical “single-compound, single-target” paradigm toward a systems-level pharmacological framework. Within this context, structurally complex natural metabolites are increasingly recognized as pleiotropic agents capable of modulating multiple, interdependent oncogenic pathways. Across diverse mechanistic domains—including mitochondrial metabolism, cytoskeletal dynamics, DNA integrity and transcriptional regulation, signal transduction cascades, epigenetic modulation, and tumor–immune microenvironment interactions—a unifying principle emerges: natural products predominantly function as multi-target modulators rather than highly selective single-site inhibitors. The well-known anticancer mechanisms of action of natural compounds are depicted in Figure 4.

This intrinsic polypharmacology is frequently rationalized in terms of the evolutionary origins of natural products, wherein ecological selection pressures favored chemical entities capable of perturbing multiple biological systems simultaneously. While this framework provides a compelling explanatory model, it should be interpreted with caution, as such multi-target activity may also arise from inherent chemical promiscuity rather than adaptive optimization for pharmacological specificity. Notwithstanding these considerations, polypharmacology may confer therapeutic advantages in the context of cancer, a disease characterized by extensive molecular heterogeneity and adaptive resistance. However, this same property introduces significant challenges, including increased potential for off-target toxicity, limited predictability of pharmacodynamic responses, and persistent difficulties in elucidating precise mechanisms of action.

A notable conceptual advancement in recent years is the recognition that the aforementioned mechanistic domains do not operate as discrete entities but rather constitute interconnected nodes within a dynamic oncogenic network. For example, mitochondrial dysfunction induced by natural products extends beyond perturbations in bioenergetics, frequently amplifying reactive oxygen species (ROS)-mediated DNA damage and reinforcing intrinsic apoptotic signaling pathways. Similarly, epigenetic modulators can indirectly influence canonical signaling cascades, such as PI3K/Akt and NF-κB, through transcriptional reprogramming, thereby functionally integrating chromatin remodeling with signal transduction. Collectively, these observations support the interpretation that the anticancer activity of natural products is more accurately conceptualized within a network pharmacology paradigm. Nevertheless, it is important to acknowledge that such network-based models remain largely descriptive, with limited capacity to delineate causality or quantify the relative contributions of individual molecular targets.

Recent advances in the exploration of marine and microbial biodiversity have further expanded the perceived boundaries of the druggable proteome, underscoring the capacity of natural products to access non-canonical binding sites and allosteric regulatory domains. Compounds targeting cytoskeletal components, particularly microtubule-associated agents, exemplify how structurally complex metabolites can engage distinct binding pockets within well-characterized protein systems. Likewise, DNA-interacting agents capable of sequence-dependent modulation of transcription illustrate alternative mechanisms for achieving selective anticancer effects without inducing indiscriminate genotoxicity. However, it is noteworthy that many such interactions occur within established target classes, suggesting that the principal innovation resides in binding modality and molecular recognition rather than the identification of entirely novel biological targets. Concurrently, advances in computational chemistry, fragment-based screening, and artificial intelligence-driven drug design are increasingly enabling synthetic compounds to access analogous non-canonical binding landscapes, thereby diminishing the exclusivity traditionally attributed to natural products in this domain.

The growing emphasis on modulation of the tumor microenvironment and immune response represents a further critical development. In contrast to earlier generations of cytotoxic agents that primarily targeted tumor cell-intrinsic processes, many contemporary natural products exhibit the capacity to reprogram the tumor ecosystem. This includes regulation of macrophage polarization, enhancement of cytotoxic T lymphocyte infiltration, remodeling of cytokine networks, and modulation of immune checkpoint expression. Such activities may facilitate the conversion of immunologically “cold” tumors into “hot” phenotypes, thereby enhancing responsiveness to immunotherapeutic interventions. However, these immunomodulatory properties are not entirely unprecedented; rather, their significance has been amplified by recent advances in immuno-oncology and the availability of more sophisticated analytical methodologies. Compared to highly specific immunotherapeutic modalities, such as immune checkpoint inhibitors and engineered cellular therapies, natural products typically exert broader but less precisely controlled effects, reflecting an inherent trade-off between systemic modulation and target specificity.

Taken together, these considerations indicate that natural products should not be viewed as a replacement for reductionist, target-based drug discovery approaches, but rather as complementary components within an increasingly integrative therapeutic paradigm. While their structural complexity and polypharmacological characteristics provide unique opportunities for addressing the multifactorial nature of cancer, substantial challenges remain with respect to specificity, mechanistic resolution, and clinical translation. Future progress in this field will likely depend on the development of hybrid strategies that integrate natural product scaffolds with advances in medicinal chemistry, systems biology, and computational modeling. Such approaches may enable the rational exploitation of polypharmacology, transforming it from an intrinsic property into a deliberately optimized feature of next-generation anticancer therapeutics.

Despite these advances, a persistent challenge across all mechanistic classes is the translational gap between preclinical efficacy and clinical success. The majority of newly identified natural products remain restricted to in vitro or early in vivo validation due to several limiting factors, including low natural abundance, structural complexity, suboptimal pharmacokinetic properties, and narrow therapeutic indices. Marine-derived macrolides and enediynes exemplify this bottleneck, wherein exceptional cytotoxic potency is counterbalanced by synthetic inaccessibility and systemic toxicity. Similarly, epigenetic and signaling modulators frequently exhibit limited target selectivity and metabolic instability, thereby constraining their standalone clinical utility [1,131]. Notably, in the past five years, only a very limited number of FDA-first global anticancer approvals (2021–2026) can be classified as natural-product-derived, first-in-class therapeutic breakthroughs, and these are almost exclusively represented by ADCs rather than conventional small-molecule cytotoxics or directly administered natural extracts [132].

However, recent technological innovations are beginning to mitigate these constraints. The integration of genome mining, metagenomics, and artificial intelligence-guided compound prioritization has substantially expanded the accessible natural product chemical space, enabling the prediction and prioritization of biosynthetic gene clusters with high therapeutic potential. In parallel, synthetic biology platforms and heterologous expression systems are addressing supply limitations by enabling scalable production of otherwise inaccessible metabolites. Moreover, the emergence of ADCs based on marine peptide scaffolds (e.g., auristatins) illustrates how structural optimization combined with targeted delivery strategies can successfully transform highly potent yet toxic natural products into clinically viable therapeutics.

From a pharmacological perspective, one of the most important emerging trends is the shift toward context-dependent cytotoxicity, in which natural products selectively exploit cancer-specific vulnerabilities such as mitochondrial dependency, replication stress, and inflammatory signaling addiction. This selectivity is particularly evident in cancer stem cell and metastatic populations, which often rely on oxidative phosphorylation and are therefore especially sensitive to mitochondrial disruptors. Similarly, tumors characterized by constitutive activation of NF-κB or PI3K/Akt signaling display increased susceptibility to natural products targeting these pathways, supporting the concept of synthetic lethality-like interactions in natural product pharmacology.

Collectively, the evidence reviewed supports a conceptual redefinition of natural products not merely as “drug leads,” but as evolutionarily optimized, multi-dimensional pharmacological systems. Their therapeutic relevance arises not only from intrinsic bioactivity but also from their capacity to engage biological networks across multiple hierarchical levels, including metabolic, genetic, epigenetic, and immunological regulation. This systems-level functionality positions natural products as uniquely suited for modern oncology, particularly in combination strategies involving immunotherapies, kinase inhibitors, and advanced drug delivery systems.

Looking forward, the field is likely to be shaped by three converging trajectories. First, artificial intelligence-driven multi-omics integration will enable predictive mapping of compound–target–pathway relationships with unprecedented resolution. Second, precision engineering of biosynthetic pathways will facilitate the rational redesign of natural scaffolds to optimize potency, selectivity, and pharmacokinetic performance. Third, clinical translation through combination therapy frameworks—particularly involving immune checkpoint inhibitors and metabolic modulators—will likely define the next generation of natural product-derived anticancer strategies.

## 4. Methods

**Study Design**

This narrative review was undertaken to systematically synthesize and critically appraise contemporary literature concerning natural products with anticancer properties. The methodological framework adhered to established standards for narrative reviews, with primary guidance derived from the SANRA (Scale for the Assessment of Narrative Review Articles) guidelines, thereby ensuring methodological transparency, rigor, and structured reporting. Furthermore, principles of reproducible evidence synthesis in narrative reviews were incorporated, including explicit documentation of search procedures, predefined eligibility criteria, and structured qualitative synthesis approaches [133,134,135]. Reporting was aligned with contemporary best-practice recommendations for narrative evidence synthesis, rather than systematic review frameworks such as PRISMA, reflecting the interpretative and conceptually integrative objectives of the present work.

**Information Sources**

A comprehensive literature search was conducted across the PubMed, Scopus, and Web of Science databases.

**Search Strategy**

The search strategy integrated controlled vocabulary and free-text keywords, combined using Boolean operators (AND, OR). The core search string was constructed as follows and adapted appropriately for each database: (“natural products” OR “naturally derived compounds” OR phytochemicals OR “secondary metabolites” OR “marine natural products” OR “microbial metabolites” OR “plant-derived compounds”) AND (cancer OR anticancer OR tumor OR neoplasia OR oncology OR “cancer therapy”) AND (“mechanism of action” OR “drug discovery” OR “drug development” OR pharmacology OR “signaling pathways” OR epigenetics OR “molecular targets”).

**Database-Specific Adaptations**

Search strategies were tailored as follows:

- PubMed: Medical Subject Headings (MeSH) terms were incorporated to improve indexing sensitivity.
- Scopus: TITLE-ABS-KEY field restrictions were applied.
- Web of Science: Topic (TS) field queries were used.

**Time Frame and Language Restrictions**

All searches were limited to peer-reviewed English-language publications published between January 2021 and March 2026.

**Study Identification and Selection Process**

**Figure 5:**
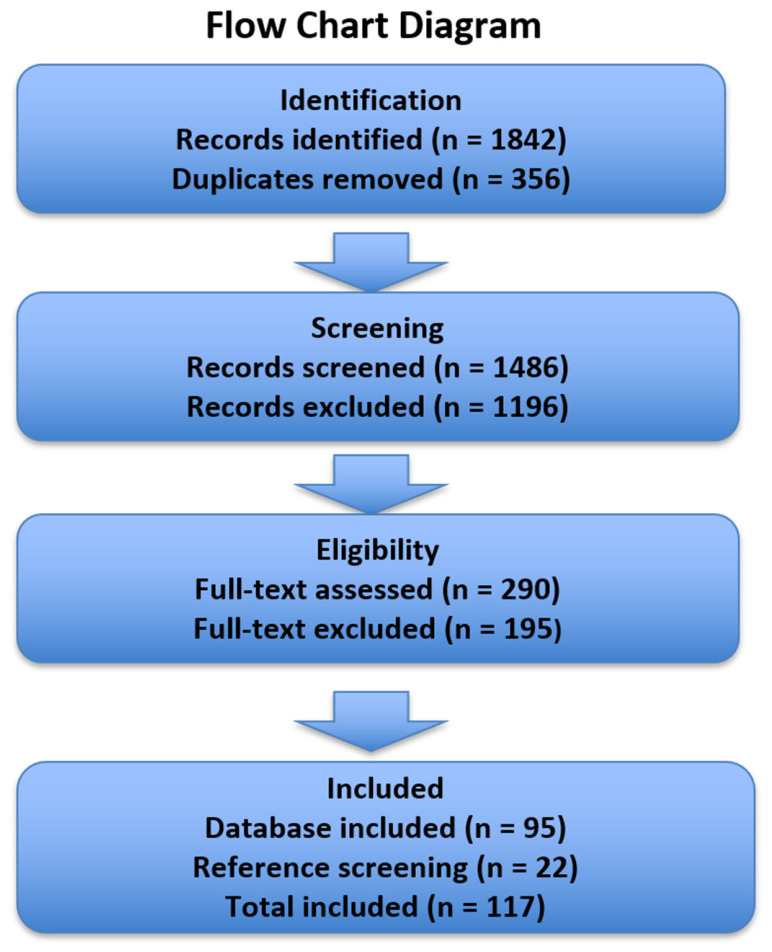
The flow chart diagram of study enrollment.

The initial search strategy identified 1842 records across all databases. Following removal of duplicate entries (n = 356), 1486 records remained eligible for screening. Title and abstract screening resulted in exclusion of non-relevant studies, yielding 290 full-text articles assessed for eligibility. Of these, 95 studies satisfied the predefined inclusion criteria through database screening. An additional 22 studies were identified via manual searching of reference lists, culminating in a final dataset comprising 117 studies included in the qualitative synthesis [136]. In Figure 5, the flow chart diagram of study enrollment is depicted.

**Screening and Eligibility Criteria**

Study selection was conducted through a two-stage screening process, encompassing title/abstract screening followed by full-text evaluation, in accordance with predefined eligibility and exclusion criteria.

**Inclusion criteria:**

1. Peer-reviewed original research articles or high-quality review papers
2. English-language publications
3. Studies reporting natural products with experimentally validated or strongly substantiated anticancer activity
4. Studies addressing chemical characterization, biological effects, molecular mechanisms, or translational relevance

**Exclusion criteria:**

1. Studies unrelated to cancer
2. Insufficient methodological or experimental detail
3. Conference abstracts, editorials, commentaries, or theses without full-text availability
4. Duplicate or redundant datasets

**Data Extraction**

Data extraction was performed on 1 May 2026 using a standardized data extraction template to ensure consistency and reproducibility. Extracted variables included:

- Author(s)
- Year of publication
- Natural product origin (plant, marine, microbial, or fungal sources)
- Compound class
- Experimental model systems (in vitro, in vivo, in silico)
- Molecular targets and signaling pathways
- Principal findings

The extracted data were subsequently organized into mechanistically and biosource-oriented thematic categories.

**Data Synthesis and Analytical Framework**

Data synthesis followed a qualitative narrative approach, whereby included studies were stratified according to principal anticancer mechanisms, including apoptosis induction, modulation of oncogenic signaling cascades, epigenetic regulation, immune system modulation, and metabolic reprogramming. A structured thematic synthesis approach was employed to enable conceptual clustering, cross-study comparison, and identification of mechanistic convergence and divergence.

**Quality Appraisal Approach**

Although no formal risk-of-bias assessment tool was applied, study quality, reproducibility, and experimental rigor were systematically considered during interpretation and synthesis. This included evaluation of experimental design robustness, adequacy of controls, and biological plausibility of findings.

**Methodological Reporting Framework**

The overall reporting strategy was informed by SANRA guidelines to ensure methodological transparency and completeness appropriate for narrative reviews, while explicitly acknowledging that PRISMA guidelines were not applied due to the non-systematic and interpretative nature of the review design.

## 5. Conclusions

In conclusion, the period spanning the last five years represents not merely an expansion in the catalog of anticancer natural products, but a qualitative transformation in their conceptualization within oncology drug discovery. Rather than functioning as isolated cytotoxic scaffolds, natural products are increasingly recognized as network-active modulators capable of simultaneously perturbing metabolic, epigenetic, signaling, and immunological hierarchies that sustain tumor viability. This shift reflects a broader evolution in cancer pharmacology—from reductionist single-target inhibition toward systems-level intervention in dynamic tumor ecosystems.

Concurrently, the field is transitioning from serendipitous discovery toward data-driven and biosynthetically engineered discovery frameworks, integrating genome mining, artificial intelligence-assisted prioritization, and synthetic biology to overcome historical limitations in accessibility, scalability, and reproducibility. However, this conceptual and technological maturation also reveals a critical tension: while mechanistic understanding and chemical diversity are advancing rapidly, clinical translation remains comparatively slow, constrained by pharmacokinetic complexity, structural intractability, and insufficient stratification of tumor contexts.

Therefore, the future impact of natural products will depend less on the identification of additional bioactive molecules and more on the systematic alignment of their multi-target pharmacology with defined cancer vulnerabilities and clinically actionable therapeutic architectures, particularly in combination with immunotherapy and precision-targeted delivery systems.

## Figures and Tables

**Table 1:** Anticancer activities of representative natural products over the last five years.

Anticancer Mechanisms	Representative Natural Products	Representative References
Mitochondrial metabolism targeting	Berberine, Resveratrol, Isobavachalcone, Hirsutine	[36,37,38]
Microtubule dynamics modulation	Paclitaxel, Colchicine, Vincristine derivatives	[39,40,41]
DNA interaction and transcriptional disruption	Doxorubicin, Camptothecin derivatives, Discorhabdin P, Dynobactin A	[1,42,43]
Signal transduction inhibition (PI3K/Akt, NF-κB, MAPK)	Curcumin, Epigallocatechin gallate (EGCG), Isobavachalcone	[44,45,46]
Epigenetic regulation and enzyme inhibition	Trichostatin A, Vorinostat-like natural analogues, Sulforaphane	[47,48,49]
Immune modulation and tumor microenvironment targeting	Polysaccharides (e.g., lentinan), β-glucans, marine-derived immunomodulatory metabolites (e.g., Erythrosponids A–C)	[50,51,52]

**Table 2:** Mitochondrial targeting-associated anticancer outcomes induced by natural products.

Mitochondrial Targeting-Associated Anticancer Mechanisms of Action	Representative Reference
ATP depletion and metabolic collapse due to OXPHOS inhibition	[66]
ROS overproduction and oxidative stress-induced apoptosis	[33]
Activation of intrinsic apoptotic pathways via cytochrome c release	[6]
Metabolic reprogramming failure in OXPHOS-dependent tumors	[54]
Synergistic vulnerability in drug-resistant and metastatic cancer cells	[36]

**Table 3:** Key findings, representative compounds, and mechanisms of natural products targeting DNA and transcriptional machinery in cancer.

Section	Key Findings (Bullet Points)	Representative Compounds/Mechanisms	References
DNA and Transcription as Targets	Cancer cells exhibit replication stress and transcriptional dependence. DNA-binding drugs remain clinically validated anticancer agents Limitations: toxicity and resistance New natural products target DNA, transcription factors, and RNA polymerases	Anthracyclines, bleomycins, minor groove binders	[1,6,10,42,43,79]
Marine DNA Minor Groove Binders	Trabectedin binds DNA minor groove and distorts helix Disrupts transcription factor binding Induces DNA breaks via transcription-coupled repair Analogues aim to improve selectivity and reduce toxicity Bryostatins indirectly regulate transcription via PKC signaling	Trabectedin (ET-743), ecteinascidins, bryostatins	[80,82,83,84,85,86]
DNA-Damaging Natural Products	Induce double-strand DNA breaks Act via radical formation or ROS generation Extremely potent at picomolar–nanomolar levels Enabled development of ADC payloads Cause replication fork collapse and apoptosis	Calicheamicin analogues, enediyne-like compounds	[87,88,99]
Epigenetic and Chromatin Modulation	Shift toward epigenetic regulation mechanisms HDAC inhibition leads to chromatin relaxation Reactivates tumor suppressor genes Some compounds inhibit RNA polymerase II Causes global transcriptional shutdown in cancer cells	HDAC inhibitors, microbial transcription inhibitors	[89,90,91]
Indirect Transcriptional Inhibition	Many natural products act via signaling pathways rather than direct DNA binding Target pathways: NF-κB, STAT3, PI3K/Akt Lead to suppression of survival gene transcription Marine alkaloids and chalcones are representative examples Advantage: lower genotoxicity vs. DNA intercalators	Marine alkaloids, chalcone derivatives	[92,93,94]

**Table 4:** Key findings, representative compounds, and mechanisms of natural products targeting oncogenic signal transduction pathways.

Section	Key Findings (Bullet Points)	Representative Compounds/Mechanisms	References
PI3K/Akt/mTOR Inhibition	Central regulator of cell survival, metabolism, proliferation Natural products inhibit PI3K phosphorylation and Akt activation Leads to mTOR suppression, apoptosis, cell-cycle arrest Compounds modulate autophagy and metabolic stress responses Often act as partial inhibitors → reduced toxicity Can reverse chemoresistance and enhance immune responses	Quercetin, berberine, curcumin, ginsenosides	[44,45,46,100,102,104]
NF-κB Pathway Inhibition	NF-κB regulates inflammation, survival, metastasis genes Frequently constitutively active in cancers Natural products inhibit IKK phosphorylation and p65 translocation Downregulates BCL-XL, XIAP, TNF-α, IL-6 Promotes apoptosis and anti-inflammatory effects Modulates tumor microenvironment and angiogenesis	Luteolin, apigenin, phenolic acids, sesquiterpene lactones	[44,45,46,102,103]
MAPK/ERK Pathway Modulation	Controls proliferation, differentiation, survival Dysregulated via Ras/BRAF mutations Natural products inhibit ERK phosphorylation and MEK activity Block cyclin D1 and c-Myc transcription Many compounds show dual/multi-pathway inhibition Helps overcome drug resistance and pathway redundancy	Oridonin, honokiol, resveratrol derivatives	[44,45,97,102]
Pathway Crosstalk and Systems-Level Modulation	Strong crosstalk between PI3K/Akt, NF-κB, MAPK Compensation leads to drug resistance Natural products exhibit multi-pathway inhibition simultaneously Induce apoptosis via mitochondrial and transcriptional mechanisms Represent systems-level therapeutic strategy	Multi-target phytochemicals and microbial metabolites	[44,45,101,102]
Therapeutic Implications and Future Directions	Limitations: poor bioavailability, instability, off-target effects Solutions: nanodelivery, prodrugs, semi-synthesis Emerging trends: – AI-driven drug discovery – Multi-omics pathway mapping – Combination therapies (immunotherapy + kinase inhibitors) – Optimization of multi-target scaffolds	Nanocarriers, optimized natural derivatives	[45,46]

**Table 5:** Key immunological findings, mechanisms of action, and immune targets affected by natural products in cancer therapy.

Section	Key Immunological Focus in TME	Mechanisms of Action	Immune Targets Affected	Natural Product Classes Mentioned	References
Innate immune suppression	Immunosuppressive myeloid compartment (TAMs, MDSCs)	Reprogramming M2 → M1 macrophages; inhibition of STAT3/NF-κB in MDSCs; reduced arginase activity and immunosuppressive cytokines (IL-10, TGF-β)	TAM polarization, MDSC expansion, CD8^+^ T cell suppression	Polysaccharides, flavonoids, alkaloids, triterpenoids	[50,51,114]
Adaptive immunity and checkpoint regulation	Dysfunctional CD8^+^ T cells, Tregs, PD-1/PD-L1 axis	Enhanced TCR signaling, increased IL-2/IFN-γ, improved CTL cytotoxicity; PD-L1 downregulation; synergy with checkpoint blockade	CD8^+^ T cells, Tregs, PD-1/PD-L1 signaling	Polyphenols (e.g., rosmarinic acid), flavonoids	[52,112,113]
Cytokine network reprogramming	Tumor-promoting inflammatory milieu (IL-6, TNF-α, IL-10)	Broad suppression/modulation of cytokine signaling; shift from pro-tumoral inflammation to immune activation; improved dendritic cell maturation and antigen presentation	IL-1β, IL-6, IL-12, TNF-α, dendritic cells	Flavonoids, terpenoids, alkaloids	[51,114,115,116]
TME remodeling and immune infiltration	Physical and cellular tumor microenvironment barriers	ECM remodeling, fibrosis reduction, angiogenesis inhibition (VEGF suppression), enhanced lymphocyte infiltration, reduced Treg recruitment	CD8^+^ T cells, Tregs, tumor vasculature, stromal ECM	Broad natural product classes (unspecified; likely polyphenols/terpenoids)	[113,114]
Systems-level immune modulation	Multi-pathway immune suppression redundancy in TME	Simultaneous modulation of macrophage polarization, T cell activation, cytokines, checkpoint expression, and stromal signaling; network-level immune reprogramming	Multiple immune compartments (innate + adaptive + stromal)	Diverse natural products across major phytochemical classes	[51,112,113,114]

## Data Availability

No new data were created or analyzed in this study.

## Abbreviations

The following abbreviations are used in this manuscript:

NMR	Nuclear Magnetic Resonance
HRMS	High-Resolution Mass Spectrometry
cryo-EM	cryo-Electron Microscopy
BGCs	Biosynthetic Gene Clusters
AI	Artificial Intelligence
ML	Machine Learning
RiPPs	Ribosomally synthesized and Post-translationally modified Peptides
NF-κB	Nuclear Factor-κΒ
MAPK	Mitogen-Activated Protein Kinase
OXPHOS	OXidative PHOsphorylation
ETC	Electron Transport Chain
ROS	Reactive Oxygen Species
NADH	Nicotinamide Adenine Dinucleotide
ATP	Adenosine TriPhosphate
MTAs	Microtubule-Targeting Agents
MMAE	MonoMethyl Auristatin E
PI3K	PhosphoInositide 3-Kinase
ADCs	Antibody–Drug Conjugates
PKC	Protein Kinase C
HDAC	Histone DeACetylase
STAT3	Signal Transducer and Activator of Transcription 3
IKK	IκB Kinase
TNF-α	Tumor Necrosis Factor-α
IL-6	Interleukin-6
MEK1/2	Mitogen-Activated Protein Kinase Kinases 1 and 2
ERK	Extracellular Signal-Regulated Kinase
mTOR	mechanistic Target Of Rapamycin
XIAP	X-linked Inhibitor of Apoptosis Protein
DNMTs	DNA MethylTransferases
HMTs	Histone MethylTransferases
EGCG	EpiGalloCatechin Gallate
BRCA1	BReast CAncer gene 1
SAM	S-AdenosylMethionine
HATs	Histone AcetylTransferases
lncRNAs	long non-coding RNAs
miRNAs	microRNA
TME	Tumor MicroEnvironment
Tregs	regulatory T cells
TAMs	Tumor-Associated Macrophages
MDSCs	Myeloid-Derived Suppressor Cells
TGF-β	Tumor Growth Factor-β
TCR	T Cell Receptor
IFN-γ	Interferon-γ
VEGF	Vascular Endothelial Growth Factor
ECM	ExtraCellular Matrix
COX-2	Cyclooxygenase-2
EMT	Epithelial–Mesenchymal Transition
PDL1	Programmed Death-Ligand 1
SANRA	Scale for the Assessment of Narrative Review Articles
MeSH	Medical Subject Headings
